# Supplementary material for: The evolution of trait variance creates a tension between species diversity and functional diversity
Source: Nat Commun. 2022 May 9;13:2521. doi: 10.1038/s41467-022-30090-4 (PMC9085882; doi:10.1038/s41467-022-30090-4)
Supplement: Supplementary file 1 — Supplementary Information [file 41467_2022_30090_MOESM1_ESM.pdf]

The evolution of trait variance creates a tension between species diversity and functional diversity

Supplementary Information

Supplementary Notes 1-8

Supplementary Note 1: Quantitative genetics of multidimensional traits **2**
Supplementary Note 1.1: Basic relationships 2
Supplementary Note 1.2: Midparent-offspring regression without selection 3
Supplementary Note 1.3: Selection on the parents 4

Supplementary Note 2: Community dynamics **4**

Supplementary Note 3: The consumer-resource model **8**

Supplementary Note 4: Eco-evolutionary dynamics in the consumer-resource model **10**

Supplementary Note 5: Calculating diversity **14**
Supplementary Note 5.1: Discrete diversity 14
Supplementary Note 5.2: Functional diversity in continuous trait spaces 14
Supplementary Note 5.3: Distance-dependent functional diversity metrics 16

Supplementary Note 6: Species- and functional diversity in model communities **17**
Supplementary Note 6.1: Simulation protocol 17
Supplementary Note 6.2: More intrinsic growth shapes in one trait dimension 18
Supplementary Note 6.3: Ecosystem functioning 18
Supplementary Note 6.4: Relaxing the assumption of Gaussian competition 21

Supplementary Note 7: Species- and functional diversity in the empirical snail communities **25**
Supplementary Note 7.1: General methodology 25
Supplementary Note 7.2: The Galápagos land snail data 26

Supplementary Note 8: Distance-dependent functional diversity metrics **27**

Supplementary Note 1 Quantitative genetics of multidimensional traits

Supplementary Note 1.1 Basic relationships

Let an individual's multidimensional phenotype $\boldsymbol{p}$ be an L-component vector, each entry holding the value of the corresponding character for that individual. We assume random mating, equal sex ratios, and purely additive genetic variation without genotype-environment interactions, dominance, epistasis, or pleiotropy. The multivariate normal distribution $\mathcal{N}(\boldsymbol{z}; \boldsymbol{\mu}, \boldsymbol{P})$ with mean $\boldsymbol{\mu}$, covariance matrix $\boldsymbol{P}$, and independent variable $\boldsymbol{z}$ is written

$$\mathcal{N}(\boldsymbol{z}; \boldsymbol{\mu}, \boldsymbol{P}) = \sqrt{\frac{1}{(2\pi)^L \det(\boldsymbol{P})}} \exp\left(-\frac{1}{2}(\boldsymbol{z} - \boldsymbol{\mu})\boldsymbol{P}^{-1}(\boldsymbol{z} - \boldsymbol{\mu})\right) \quad (1)$$

(see Supplementary Table 1 at the end of this document for the list of all symbols and their meaning). The phenotype $\boldsymbol{p}$ is composed of independent genetic and environmental factors, each of which is assumed to be sampled from an L-normal distribution:

$$\boldsymbol{p} = \boldsymbol{m} + \boldsymbol{f} + \boldsymbol{e}, \quad (2)$$

where $\boldsymbol{m}$ and $\boldsymbol{f}$ are the genetic contribution of the individual's mother and father, and $\boldsymbol{e}$ is the environmental contribution. While this description is purely statistical, it helps to think of the genetic component in the following way: each entry of $\boldsymbol{m}$ and $\boldsymbol{f}$ represents a single locus involved in coding for the corresponding trait, each locus having very many possible alleles.

The variance-covariance matrix $\mathrm{cov}(\boldsymbol{p})$ of the phenotype vector is calculated as

$$\begin{aligned} \mathrm{cov}(\boldsymbol{p}) &= \mathrm{cov}(\boldsymbol{m} + \boldsymbol{f} + \boldsymbol{e}) \\ &= \mathrm{cov}(\boldsymbol{m}) + \mathrm{cov}(\boldsymbol{f}) + \mathrm{cov}(\boldsymbol{e}) + 2\mathrm{cov}(\boldsymbol{m}, \boldsymbol{f}) + 2\mathrm{cov}(\boldsymbol{m}, \boldsymbol{e}) + 2\mathrm{cov}(\boldsymbol{f}, \boldsymbol{e}), \end{aligned} \quad (3)$$

where $\mathrm{cov}(\boldsymbol{x}, \boldsymbol{y})$ is the cross-covariance between two vectors $\boldsymbol{x}$ and $\boldsymbol{y}$. Since $\boldsymbol{m}$ and $\boldsymbol{f}$ stem from independently derived alleles, $\mathrm{cov}(\boldsymbol{m}, \boldsymbol{f}) = 0$. Similarly, due to the lack of genotype-environment interactions, the other two cross-covariance terms are zero as well. This leaves

$$\mathrm{cov}(\boldsymbol{p}) = \mathrm{cov}(\boldsymbol{m}) + \mathrm{cov}(\boldsymbol{f}) + \mathrm{cov}(\boldsymbol{e}). \quad (4)$$

We will denote $\mathrm{cov}(\boldsymbol{m}) = \mathrm{cov}(\boldsymbol{f})$ by $\boldsymbol{G}/2$, where $\boldsymbol{G}$ is the additive genetic variance-covariance matrix. Similarly, we write $\boldsymbol{E} = \mathrm{cov}(\boldsymbol{e})$ and $\boldsymbol{P} = \mathrm{cov}(\boldsymbol{p})$. Supplementary Eq. 4 then reads

$$\boldsymbol{P} = \boldsymbol{G} + \boldsymbol{E}, \quad (5)$$

with $\boldsymbol{P}$ being the sum of the additive genetic and environmental covariance matrices.

Next, we determine the covariance between a single parent and her offspring. Let their phenotypes be

$$\boldsymbol{p}_p = \boldsymbol{m}_p + \boldsymbol{f}_p + \boldsymbol{e}_p, \qquad \boldsymbol{p} = \boldsymbol{m}_p + \boldsymbol{f} + \boldsymbol{e}, \quad (6)$$

where the subscript p denotes parental quantities. The maternal allele of the offspring, for each trait, is received from its mother, therefore $\boldsymbol{m} = \boldsymbol{m}_p$ was used above. The cross-covariance reads

$$\begin{aligned} \mathrm{cov}(\boldsymbol{p}_p, \boldsymbol{p}) = \mathrm{cov}(\boldsymbol{m}_p + \boldsymbol{f}_p + \boldsymbol{e}_p, \boldsymbol{m}_p + \boldsymbol{f} + \boldsymbol{e}) = \mathrm{cov}(\boldsymbol{m}_p) + \mathrm{cov}(\boldsymbol{m}_p, \boldsymbol{f}) + \mathrm{cov}(\boldsymbol{f}_p, \boldsymbol{m}_p) \\ + \mathrm{cov}(\boldsymbol{f}_p, \boldsymbol{f}) + \mathrm{cov}(\boldsymbol{m}_p, \boldsymbol{e}) + \mathrm{cov}(\boldsymbol{f}_p, \boldsymbol{e}) + \mathrm{cov}(\boldsymbol{e}_p, \boldsymbol{m}_p) + \mathrm{cov}(\boldsymbol{e}_p, \boldsymbol{f}) + \mathrm{cov}(\boldsymbol{e}_p, \boldsymbol{e}). \end{aligned} \quad (7)$$

The first term is $\mathrm{cov}(\boldsymbol{m}_p) = \mathrm{cov}(\boldsymbol{m}) = \boldsymbol{G}/2$. Terms 2-4 are zero because they are cross-covariances between independently derived alleles. Terms 5-8 are also zero due to the absence of genotype-environment interactions. Finally, we assume that the correlation between parent and offspring environments is negligible, setting $\mathrm{cov}(\boldsymbol{e}_p, \boldsymbol{e})$ to zero. In the end, we have

$$\mathrm{cov}(\boldsymbol{p}_p, \boldsymbol{p}) = \frac{1}{2}\boldsymbol{G}. \quad (8)$$

This turns out to be the same as the midparent-offspring covariance. The midparent phenotype reads $\boldsymbol{p}_m = (\boldsymbol{p}_1 + \boldsymbol{p}_2)/2$, from which

$$\mathrm{cov}(\boldsymbol{p}_m, \boldsymbol{p}) = \mathrm{cov}\Big(\frac{\boldsymbol{p}_1 + \boldsymbol{p}_2}{2}, \boldsymbol{p}\Big) = \frac{1}{2}\Big[\underbrace{\mathrm{cov}(\boldsymbol{p}_1, \boldsymbol{p})}_{\boldsymbol{G}/2} + \underbrace{\mathrm{cov}(\boldsymbol{p}_2, \boldsymbol{p})}_{\boldsymbol{G}/2}\Big] = \frac{1}{2}\boldsymbol{G}. \tag{9}$$

The variance-covariance matrix of midparent phenotype reads

$$\mathrm{cov}(\boldsymbol{p}_m) = \mathrm{cov}\Big(\frac{\boldsymbol{p}_1 + \boldsymbol{p}_2}{2}\Big) = \frac{1}{4}\underbrace{\mathrm{cov}(\boldsymbol{p}_1)}_{\boldsymbol{P}} + \frac{1}{4}\underbrace{\mathrm{cov}(\boldsymbol{p}_2)}_{\boldsymbol{P}} + \frac{1}{2}\underbrace{\mathrm{cov}(\boldsymbol{p}_1, \boldsymbol{p}_2)}_{0} = \frac{1}{2}\boldsymbol{P}. \tag{10}$$

Supplementary Note 1.2 Midparent-offspring regression without selection

To perform the regression of offspring on midparents, we construct a $2L$-dimensional multinormal distribution whose first L dimensions correspond to midparents, and the second L to offspring. Its mean $\boldsymbol{\mu}_{p_m,p}$ and covariance matrix $\boldsymbol{\Sigma}_{p_m,p}$ can be written in block-matrix form:

$$\boldsymbol{\mu}_{p_m,p} = \begin{pmatrix}\boldsymbol{\mu}\\ \boldsymbol{\mu}\end{pmatrix}, \qquad \boldsymbol{\Sigma}_{p_m,p} = \begin{pmatrix}\mathrm{cov}(\boldsymbol{p}_m) & \mathrm{cov}(\boldsymbol{p}_m, \boldsymbol{p})\\ \mathrm{cov}(\boldsymbol{p}, \boldsymbol{p}_m) & \mathrm{cov}(\boldsymbol{p})\end{pmatrix}, \tag{11}$$

where the mean of midparents and offspring are equal due to the lack of selection (note also that the midparent and individual parental mean phenotypes are equal). Using $\boldsymbol{P} = \mathrm{cov}(\boldsymbol{p})$ and Supplementary Eqs. 9-10, the covariance terms above can be written as

$$\boldsymbol{\Sigma}_{p_m,p} = \begin{pmatrix}\boldsymbol{P}/2 & \boldsymbol{G}/2\\ \boldsymbol{G}/2 & \boldsymbol{P}\end{pmatrix}. \tag{12}$$

Using standard multivariate regression [1], the expected offspring phenotype $\boldsymbol{\mu}'$ given a particular midparent phenotype $\boldsymbol{x}$ is

$$\boldsymbol{\mu}'(\boldsymbol{x}) = \mathrm{cov}(\boldsymbol{p}_m, \boldsymbol{p})\mathrm{cov}(\boldsymbol{p}_m)^{-1}(\boldsymbol{x} - \boldsymbol{\mu}) + \boldsymbol{\mu} = \boldsymbol{G}\boldsymbol{P}^{-1}(\boldsymbol{x} - \boldsymbol{\mu}) + \boldsymbol{\mu}, \tag{13}$$

and the covariance of offspring (also known as the the segregation variance) reads

$$\boldsymbol{S} = \mathrm{cov}(\boldsymbol{p}) - \mathrm{cov}(\boldsymbol{p}_m, \boldsymbol{p})\mathrm{cov}(\boldsymbol{p}_m)^{-1}\mathrm{cov}(\boldsymbol{p}_m, \boldsymbol{p}) = \boldsymbol{P} - \frac{1}{2}\boldsymbol{G}\boldsymbol{P}^{-1}\boldsymbol{G}. \tag{14}$$

The total phenotypic covariance of offspring is composed of two parts. First, the covariance matrix of $\boldsymbol{\mu}'$. Second, since, $\boldsymbol{\mu}'$ is the expected offspring phenotype given a midparent phenotype, one has to add the segregation variance $\boldsymbol{S}$ to this result. The two are indeed additive without cross-covariance terms, since a regression is always uncorrelated with its residuals. This means that the total phenotypic covariance of offspring can be written

$$\boldsymbol{P}' = \mathrm{cov}(\boldsymbol{\mu}') + \boldsymbol{S} = \mathrm{cov}\Big(\boldsymbol{G}\boldsymbol{P}^{-1}(\boldsymbol{x} - \boldsymbol{\mu}) + \boldsymbol{\mu}\Big) + \boldsymbol{P} - \frac{1}{2}\boldsymbol{G}\boldsymbol{P}^{-1}\boldsymbol{G}. \tag{15}$$

Using the identity $\mathrm{cov}(\boldsymbol{A}\boldsymbol{x} + \boldsymbol{a}) = \boldsymbol{A}\mathrm{cov}(\boldsymbol{x})\boldsymbol{A}^{\mathrm{T}}$ (where $\boldsymbol{A}$ is a constant matrix, $\boldsymbol{A}^{\mathrm{T}}$ its transpose, $\boldsymbol{a}$ a constant vector, and $\boldsymbol{x}$ the random vector), we get

$$\begin{aligned}\boldsymbol{P}' &= \mathrm{cov}\Big(\boldsymbol{G}\boldsymbol{P}^{-1}\boldsymbol{x} - \big(\boldsymbol{G}\boldsymbol{P}^{-1}\boldsymbol{\mu} + \boldsymbol{\mu}\big)\Big) + \boldsymbol{P} - \frac{1}{2}\boldsymbol{G}\boldsymbol{P}^{-1}\boldsymbol{G}\\ &= \boldsymbol{G}\boldsymbol{P}^{-1}\mathrm{cov}(\boldsymbol{x})\boldsymbol{P}^{-1}\boldsymbol{G} + \boldsymbol{P} - \frac{1}{2}\boldsymbol{G}\boldsymbol{P}^{-1}\boldsymbol{G},\end{aligned} \tag{16}$$

where we used the fact that $(\boldsymbol{G}\boldsymbol{P}^{-1})^{\mathrm{T}} = (\boldsymbol{P}^{-1})^{\mathrm{T}}\boldsymbol{G}^{\mathrm{T}} = \boldsymbol{P}^{-1}\boldsymbol{G}$, since both $\boldsymbol{G}$ and $\boldsymbol{P}$ are covariance matrices and thus symmetric. By Supplementary Eq. 5, $\boldsymbol{P} = \boldsymbol{G} + \boldsymbol{E}$ and $\boldsymbol{P}' = \boldsymbol{G}' + \boldsymbol{E}$ (assuming a constant environmental covariance). Inserting the identity matrix $\boldsymbol{P}\boldsymbol{P}^{-1}$ into the last term:

$$\boldsymbol{P}' = \boldsymbol{G}' + \boldsymbol{E} = \boldsymbol{G}\boldsymbol{P}^{-1}\mathrm{cov}(\boldsymbol{x})\boldsymbol{P}^{-1}\boldsymbol{G} + \boldsymbol{G} + \boldsymbol{E} - \frac{1}{2}(\boldsymbol{G}\boldsymbol{P}^{-1})\boldsymbol{P}(\boldsymbol{P}^{-1}\boldsymbol{G}), \tag{17}$$

or

$$G' = GP^{-1}\left(\mathrm{cov}(x) - \frac{1}{2}P\right)P^{-1}G + G. \tag{18}$$

Supplementary Eqs. 13 and 18 can also be written more transparently in terms of the difference between values at the two generations, $\Delta\mu = \mu' - \mu$ and $\Delta G = G' - G$:

$$\Delta\mu = GP^{-1}(x - \mu), \tag{19}$$

$$\Delta G = GP^{-1}\left(\mathrm{cov}(x) - \frac{1}{2}P\right)P^{-1}G. \tag{20}$$

Supplementary Note 1.3 Selection on the parents

The regression results above hold in the absence of selection. We now consider what happens when the mean parental phenotype changes from the original μ to a new $\tilde{\mu}$ due to selection.

Supplementary Eq. 19 gives the expected phenotype change as a function of the midparent phenotype x. The mean midparent phenotype is in turn μ without selection (which is also equal to the mean parental phenotype). Without selection, substituting $x = \mu$ in Supplementary Eq. 19 predicts no expected phenotype change. With selection however, x should be replaced by $\tilde{\mu}$. Then Supplementary Eq. 19 reads

$$\Delta\mu = GP^{-1}(\tilde{\mu} - \mu), \tag{21}$$

which is the breeder's equation for multiple characters. The matrix GP^{-1} is the multidimensional analogue of the heritability $h^2 = G/P$. Note that quantities in Supplementary Eq. 21 now refer to parents instead of midparents.

The change in additive genetic covariance is given by Supplementary Eq. 20. Again, without selection this leads to no change, because

$$\Delta G = GP^{-1}\Big(\underbrace{\mathrm{cov}(\mu)}_{\frac{P}{2}\text{ (Eq. 10)}} - \frac{1}{2}P\Big)P^{-1}G = 0. \tag{22}$$

The way to incorporate the effect of selection is again to assume that the mean midparent phenotype μ has changed to $\tilde{\mu}$. Using this and Supplementary Eq. 10, this means we have to replace $\mathrm{cov}(\mu) = P/2$ with $\mathrm{cov}(\tilde{\mu}) = \tilde{P}/2$ in Supplementary Eq. 22:

$$\Delta G = \frac{1}{2}GP^{-1}(\tilde{P} - P)P^{-1}G, \tag{23}$$

where $\tilde{P}$ is the phenotypic covariance of parents after selection. Once again, the equation is now expressed through quantities pertaining to parents as opposed to midparents.

This derivation of the genetic covariance under selection tacitly assumes that the change in segregation variance S from one generation to the next can be neglected. While this will only be exact in the absence of selection, it is a good approximation unless selection is very strong [2].

Supplementary Note 2 Community dynamics

We model the dynamics of S species differing in L ecologically relevant traits, collected in the vector z. The trait distribution within each species i is then

$$p_i(z) = \mathcal{N}(z; \mu_i, P_i) \tag{24}$$

(with $\mathcal{N}(z; \mu_i, P_i)$ given by Supplementary Eq. 1), where the mean trait μ_i and phenotypic covariance matrix P_i are time-dependent. It satisfies the normalization condition

$$\int_{-\infty}^{+\infty}\int_{-\infty}^{+\infty}\cdots\int_{-\infty}^{+\infty} p_i(z)\,\mathrm{d}z^1\mathrm{d}z^2\cdots\mathrm{d}z^L = 1 \tag{25}$$

at every moment of time (superscripts show trait indices; subscripts are reserved for the species index). Using the notation $\mathrm{d}\boldsymbol{z} = \mathrm{d}z^1 \mathrm{d}z^2 \cdots \mathrm{d}z^L$, we write the integral simply as

$$\int p_i(\boldsymbol{z})\,\mathrm{d}\boldsymbol{z} = 1. \tag{26}$$

The total population density of species i is N_i. Assuming discrete non-overlapping generations, the life cycle follows

$$N_i p_i(\boldsymbol{z}) \xrightarrow{\text{selection}} \tilde{N}_i \tilde{p}_i(\boldsymbol{z}) \xrightarrow{\text{reproduction}} N_i' p_i'(z), \tag{27}$$

where tildes denote state after selection but before reproduction, and primes the state in the next generation. Let $W_i(\boldsymbol{z})$ be the fitness of species i's phenotype $\boldsymbol{z}$ at a given moment in time:

$$\tilde{N}_i \tilde{p}_i(\boldsymbol{z}) = W_i(\boldsymbol{z}) N_i p_i(\boldsymbol{z}). \tag{28}$$

$W_i(\boldsymbol{z})$ generally depends on the abundances and trait distributions of all interacting species. Here we apply the standard sleight of hand of assuming that $W_i(\boldsymbol{z})$ encodes both birth and death processes, and the only role of "reproduction" in Supplementary Eq. 27 is to restore the trait distribution's shape to normal—in other words, $\tilde{N}_i = N_i'$. The error made this way is negligible in the weak selection limit [3]. This limit means $W_i(\boldsymbol{z})$ can be written as

$$W_i(\boldsymbol{z}) = 1 + s r_i(\boldsymbol{z}), \tag{29}$$

where $s \ll 1$ is a small parameter and $r_i(\boldsymbol{z})$ is the per capita growth rate of species i, determined by the ecological interactions within the community.

The dynamics of the population densities from one generation to the next is obtained by integrating Supplementary Eq. 28 over $\boldsymbol{z}$:

$$\tilde{N}_i \int \tilde{p}_i(\boldsymbol{z})\,\mathrm{d}\boldsymbol{z} = N_i \int W_i(\boldsymbol{z}) p_i(\boldsymbol{z})\,\mathrm{d}\boldsymbol{z}. \tag{30}$$

From Supplementary Eq. 26, the integral on the left hand side is 1. Using $\tilde{N}_i = N_i'$,

$$N_i' = N_i \int W_i(\boldsymbol{z}) p_i(\boldsymbol{z})\,\mathrm{d}\boldsymbol{z}. \tag{31}$$

In the weak selection limit of Supplementary Eq. 29,

$$N_i' = N_i \int (1 + s r_i(\boldsymbol{z})) p_i(\boldsymbol{z})\,\mathrm{d}\boldsymbol{z} = N_i \left(1 + s \int r_i(\boldsymbol{z}) p_i(\boldsymbol{z})\,\mathrm{d}\boldsymbol{z} \right), \tag{32}$$

where Supplementary Eq. 26 was used again in the last step. Subtracting N_i and dividing by s gives

$$\frac{N_i' - N_i}{s} = N_i \int r_i(\boldsymbol{z}) p_i(\boldsymbol{z})\,\mathrm{d}\boldsymbol{z}, \tag{33}$$

which, with appropriate scaling and for $s \to 0$, leads to the following differential equation [3]:

$$\frac{\mathrm{d}N_i}{\mathrm{d}t} = N_i \int r_i(\boldsymbol{z}) p_i(\boldsymbol{z})\,\mathrm{d}\boldsymbol{z}. \tag{34}$$

To track the change of trait means, we use the multidimensional breeder's equation, Supplementary Eq. 21. To do so, we need to express $\tilde{\boldsymbol{\mu}}_i$. By definition, the mean of $p_i(\boldsymbol{z})$ is

$$\boldsymbol{\mu}_i = \int \boldsymbol{z} p_i(\boldsymbol{z})\,\mathrm{d}\boldsymbol{z}, \tag{35}$$

where the integral is interpreted entry-wise: $\mu_i^k = \int z^k p_i(z)\,\mathrm{d}z$. Let us rearrange Supplementary Eq. 28:

$$\tilde{p}_i(z) = \frac{W_i(z) N_i p_i(z)}{\tilde{N}_i}. \tag{36}$$

Using $\tilde{N}_i = N_i'$ and then Supplementary Eq. 31 in the denominator:

$$\tilde{p}_i(z) = \frac{W_i(z) N_i p_i(z)}{N_i \displaystyle\int W_i(z) p_i(z)\,\mathrm{d}z} = \frac{W_i(z) p_i(z)}{\displaystyle\int W_i(z) p_i(z)\,\mathrm{d}z}. \tag{37}$$

Multiplying both sides by z and integrating, we get

$$\int z \tilde{p}_i(z)\,\mathrm{d}z = \frac{\displaystyle\int z W_i(z) p_i(z)\,\mathrm{d}z}{\displaystyle\int W_i(z) p_i(z)\,\mathrm{d}z}. \tag{38}$$

Applying Supplementary Eq. 35 on the left, the integral is simply $\tilde{\boldsymbol{\mu}}_i$:

$$\tilde{\boldsymbol{\mu}}_i = \frac{\displaystyle\int z W_i(z) p_i(z)\,\mathrm{d}z}{\displaystyle\int W_i(z) p_i(z)\,\mathrm{d}z}. \tag{39}$$

Substituting this into Supplementary Eq. 21 gives

$$\Delta\boldsymbol{\mu}_i = \boldsymbol{G}_i \boldsymbol{P}_i^{-1} \left(\frac{\displaystyle\int z W_i(z) p_i(z)\,\mathrm{d}z}{\displaystyle\int W_i(z) p_i(z)\,\mathrm{d}z} - \boldsymbol{\mu}_i \right). \tag{40}$$

We again apply the weak selection limit (Supplementary Eq. 29):

$$\Delta\boldsymbol{\mu}_i = \boldsymbol{G}_i \boldsymbol{P}_i^{-1} \left(\frac{\displaystyle\int z (1 + s r_i(z)) p_i(z)\,\mathrm{d}z}{\displaystyle\int (1 + s r_i(z)) p_i(z)\,\mathrm{d}z} - \boldsymbol{\mu}_i \right), \tag{41}$$

or

$$\Delta\boldsymbol{\mu}_i = \boldsymbol{G}_i \boldsymbol{P}_i^{-1} \left(\frac{\displaystyle\int z p_i(z)\,\mathrm{d}z + s \int z r_i(z) p_i(z)\,\mathrm{d}z}{1 + s \displaystyle\int r_i(z) p_i(z)\,\mathrm{d}z} - \boldsymbol{\mu}_i \right). \tag{42}$$

The first integral in the numerator is $\boldsymbol{\mu}_i$ by Supplementary Eq. 35:

$$\Delta\boldsymbol{\mu}_i = \boldsymbol{G}_i \boldsymbol{P}_i^{-1} \left(\frac{\boldsymbol{\mu}_i + s \displaystyle\int z r_i(z) p_i(z)\,\mathrm{d}z}{1 + s \displaystyle\int r_i(z) p_i(z)\,\mathrm{d}z} - \boldsymbol{\mu}_i \right). \tag{43}$$

Taylor expanding to first order in s gives

$$\Delta\boldsymbol{\mu}_i = \boldsymbol{G}_i \boldsymbol{P}_i^{-1} \left[\boldsymbol{\mu}_i + s \left(\int z r_i(z) p_i(z)\,\mathrm{d}z - \boldsymbol{\mu}_i \int r_i(z) p_i(z)\,\mathrm{d}z \right) - \boldsymbol{\mu}_i \right] + \mathcal{O}(s^2), \tag{44}$$

where $\mathcal{O}(s^k)$ means terms proportional to the kth power of s or higher. After simplifying,

$$\Delta\boldsymbol{\mu}_i = s\, \boldsymbol{G}_i \boldsymbol{P}_i^{-1} \int (z - \boldsymbol{\mu}_i) r_i(z) p_i(z)\,\mathrm{d}z + \mathcal{O}(s^2). \tag{45}$$

Dividing by s and performing the $s \to 0$ limit gives the differential equation

$$\frac{\mathrm{d}\boldsymbol{\mu}_i}{\mathrm{d}t} = \boldsymbol{G}_i \boldsymbol{P}_i^{-1} \int (\boldsymbol{z} - \boldsymbol{\mu}_i) r_i(\boldsymbol{z}) p_i(\boldsymbol{z}) \, \mathrm{d}\boldsymbol{z}. \tag{46}$$

Finally, the change in the genetic covariance matrices $\boldsymbol{G}_i$ can be tracked via Supplementary Eq. 23. To use this equation, we first need to express $\tilde{\boldsymbol{P}}_i$. In general, the covariance matrix of a vector $\boldsymbol{x}$ reads

$$\mathrm{cov}(\boldsymbol{x}) = \int (\boldsymbol{z} - \boldsymbol{x}) \circ (\boldsymbol{z} - \boldsymbol{x}) p_i(\boldsymbol{z}) \, \mathrm{d}\boldsymbol{z}, \tag{47}$$

where $\circ$ denotes the outer product (i.e, for vectors $\boldsymbol{a}$ and $\boldsymbol{b}$ with entries a^k and b^k, the matrix $\boldsymbol{A} = \boldsymbol{a} \circ \boldsymbol{b}$ has entries $A^{kl} = a^k b^l$). As before, the integral is interpreted entry-wise: $[\mathrm{cov}(\boldsymbol{x})]^{kl} = \int (z^k - x^k)(z^l - x^l) p_i(\boldsymbol{z}) \, \mathrm{d}\boldsymbol{z}$. Based on this, $\tilde{\boldsymbol{P}}_i$ reads

$$\tilde{\boldsymbol{P}}_i = \int (\boldsymbol{z} - \tilde{\boldsymbol{\mu}}_i) \circ (\boldsymbol{z} - \tilde{\boldsymbol{\mu}}_i) \tilde{p}_i(\boldsymbol{z}) \, \mathrm{d}\boldsymbol{z}, \tag{48}$$

or, using Supplementary Eq. 37,

$$\tilde{\boldsymbol{P}}_i = \frac{\int (\boldsymbol{z} - \tilde{\boldsymbol{\mu}}_i) \circ (\boldsymbol{z} - \tilde{\boldsymbol{\mu}}_i) W_i(\boldsymbol{z}) p_i(\boldsymbol{z}) \, \mathrm{d}\boldsymbol{z}}{\int W_i(\boldsymbol{z}) p_i(\boldsymbol{z}) \, \mathrm{d}\boldsymbol{z}}. \tag{49}$$

In the weak selection limit (Supplementary Eq. 29), this is written as

$$\begin{aligned} \tilde{\boldsymbol{P}}_i &= \frac{\int (\boldsymbol{z} - \tilde{\boldsymbol{\mu}}_i) \circ (\boldsymbol{z} - \tilde{\boldsymbol{\mu}}_i)(1 + s r_i(\boldsymbol{z})) p_i(\boldsymbol{z}) \, \mathrm{d}\boldsymbol{z}}{\int (1 + s r_i(\boldsymbol{z})) p_i(\boldsymbol{z}) \, \mathrm{d}\boldsymbol{z}} \\ &= \frac{\int (\boldsymbol{z} - \tilde{\boldsymbol{\mu}}_i) \circ (\boldsymbol{z} - \tilde{\boldsymbol{\mu}}_i) p_i(\boldsymbol{z}) \, \mathrm{d}\boldsymbol{z} + s \int (\boldsymbol{z} - \tilde{\boldsymbol{\mu}}_i) \circ (\boldsymbol{z} - \tilde{\boldsymbol{\mu}}_i) r_i(\boldsymbol{z}) p_i(\boldsymbol{z}) \, \mathrm{d}\boldsymbol{z}}{1 + s \int r_i(\boldsymbol{z}) p_i(\boldsymbol{z}) \, \mathrm{d}\boldsymbol{z}}. \end{aligned} \tag{50}$$

Taylor expanding in s to linear order yields

$$\begin{aligned} \tilde{\boldsymbol{P}}_i = \int (\boldsymbol{z} - \tilde{\boldsymbol{\mu}}_i) \circ (\boldsymbol{z} - \tilde{\boldsymbol{\mu}}_i) p_i(\boldsymbol{z}) \, \mathrm{d}\boldsymbol{z} + s \bigg[\int (\boldsymbol{z} - \tilde{\boldsymbol{\mu}}_i) \circ (\boldsymbol{z} - \tilde{\boldsymbol{\mu}}_i) r_i(\boldsymbol{z}) p_i(\boldsymbol{z}) \, \mathrm{d}\boldsymbol{z} \\ - \left(\int (\boldsymbol{z} - \tilde{\boldsymbol{\mu}}_i) \circ (\boldsymbol{z} - \tilde{\boldsymbol{\mu}}_i) p_i(\boldsymbol{z}) \, \mathrm{d}\boldsymbol{z} \right) \left(\int r_i(\boldsymbol{z}) p_i(\boldsymbol{z}) \, \mathrm{d}\boldsymbol{z} \right) \bigg] + O(s^2). \end{aligned} \tag{51}$$

To make further progress, we need to express $\tilde{\boldsymbol{\mu}}_i$ via $\boldsymbol{\mu}_i$. This can be done by equating the right hand sides of Supplementary Eqs. 21 and 45, and solving for $\tilde{\boldsymbol{\mu}}$, leading to

$$\tilde{\boldsymbol{\mu}}_i = \boldsymbol{\mu}_i + s \int (\boldsymbol{z} - \boldsymbol{\mu}_i) r_i(\boldsymbol{z}) p_i(\boldsymbol{z}) \, \mathrm{d}\boldsymbol{z} + O(s^2). \tag{52}$$

As a simplifying notation, let us call the integral above $\boldsymbol{q}_i$: $\tilde{\boldsymbol{\mu}}_i = \boldsymbol{\mu}_i + s\boldsymbol{q}_i + O(s^2)$. We substitute this into Supplementary Eq. 51 and neglect higher-order terms in s. Let us first consider the first term in Supplementary Eq. 51:

$$\begin{aligned} \int (\boldsymbol{z} - \tilde{\boldsymbol{\mu}}_i) \circ (\boldsymbol{z} - \tilde{\boldsymbol{\mu}}_i) p_i(\boldsymbol{z}) \, \mathrm{d}\boldsymbol{z} &= \int (\boldsymbol{z} - \boldsymbol{\mu}_i - s\boldsymbol{q}_i) \circ (\boldsymbol{z} - \boldsymbol{\mu}_i - s\boldsymbol{q}_i) p_i(\boldsymbol{z}) \, \mathrm{d}\boldsymbol{z} + O(s^2) \\ = \underbrace{\int (\boldsymbol{z} - \boldsymbol{\mu}_i) \circ (\boldsymbol{z} - \boldsymbol{\mu}_i) p_i(\boldsymbol{z}) \, \mathrm{d}\boldsymbol{z}}_{\boldsymbol{P}_i} &- 2s \underbrace{\left(\int (\boldsymbol{z} - \boldsymbol{\mu}_i) p_i(\boldsymbol{z}) \, \mathrm{d}\boldsymbol{z} \right)}_{0} \circ \boldsymbol{q}_i + O(s^2) = \boldsymbol{P}_i + O(s^2). \end{aligned} \tag{53}$$

The first braced term is by definition the total phenotypic covariance, while the second is zero because it is the integral of an odd and an even function in $\boldsymbol{z} - \boldsymbol{\mu}_i$ (Supplementary Eq. 24). The second term in Supplementary Eq. 51 is much simpler: since this is already proportional to s, any $s\boldsymbol{q}_i$ terms will be proportional to s^2 and can be neglected. We can therefore simply omit the $s\boldsymbol{q}_i$ terms, which means replacing $\tilde{\boldsymbol{\mu}}_i$ by $\boldsymbol{\mu}_i$. The second term in Supplementary Eq. 51 therefore reads

$$\begin{aligned} s\Bigg[\int (\boldsymbol{z} - \tilde{\boldsymbol{\mu}}_i) \circ (\boldsymbol{z} - \tilde{\boldsymbol{\mu}}_i) r_i(\boldsymbol{z}) p_i(\boldsymbol{z}) \,\mathrm{d}\boldsymbol{z} - \left(\int (\boldsymbol{z} - \tilde{\boldsymbol{\mu}}_i) \circ (\boldsymbol{z} - \tilde{\boldsymbol{\mu}}_i) p_i(\boldsymbol{z}) \,\mathrm{d}\boldsymbol{z} \right)\left(\int r_i(\boldsymbol{z}) p_i(\boldsymbol{z}) \,\mathrm{d}\boldsymbol{z} \right) \Bigg] \\ = s\Bigg[\int (\boldsymbol{z} - \boldsymbol{\mu}_i) \circ (\boldsymbol{z} - \boldsymbol{\mu}_i) r_i(\boldsymbol{z}) p_i(\boldsymbol{z}) \,\mathrm{d}\boldsymbol{z} \\ - \underbrace{\left(\int (\boldsymbol{z} - \boldsymbol{\mu}_i) \circ (\boldsymbol{z} - \boldsymbol{\mu}_i) p_i(\boldsymbol{z}) \,\mathrm{d}\boldsymbol{z} \right)}_{\boldsymbol{P}_i} \left(\int r_i(\boldsymbol{z}) p_i(\boldsymbol{z}) \,\mathrm{d}\boldsymbol{z} \right) \Bigg] + O(s^2), \end{aligned} \tag{54}$$

or

$$\begin{aligned} s\left[\int (\boldsymbol{z} - \boldsymbol{\mu}_i) \circ (\boldsymbol{z} - \boldsymbol{\mu}_i) r_i(\boldsymbol{z}) p_i(\boldsymbol{z}) \,\mathrm{d}\boldsymbol{z} - \boldsymbol{P}_i \int r_i(\boldsymbol{z}) p_i(\boldsymbol{z}) \,\mathrm{d}\boldsymbol{z} \right] + O(s^2) \\ = s \int [(\boldsymbol{z} - \boldsymbol{\mu}_i) \circ (\boldsymbol{z} - \boldsymbol{\mu}_i) - \boldsymbol{P}_i] r_i(\boldsymbol{z}) p_i(\boldsymbol{z}) \,\mathrm{d}\boldsymbol{z} + O(s^2). \end{aligned} \tag{55}$$

Substituting Supplementary Eqs. 53 and 54 into Supplementary Eq. 51:

$$\tilde{\boldsymbol{P}}_i = \boldsymbol{P}_i + s \int [(\boldsymbol{z} - \boldsymbol{\mu}_i) \circ (\boldsymbol{z} - \boldsymbol{\mu}_i) - \boldsymbol{P}_i] r_i(\boldsymbol{z}) p_i(\boldsymbol{z}) \,\mathrm{d}\boldsymbol{z} + O(s^2). \tag{56}$$

Substituting this in turn into Supplementary Eq. 23, we get

$$\begin{aligned} \Delta \boldsymbol{G}_i &= \frac{1}{2} \boldsymbol{G}_i \boldsymbol{P}_i^{-1} \left[\boldsymbol{P}_i + s \int [(\boldsymbol{z} - \boldsymbol{\mu}_i) \circ (\boldsymbol{z} - \boldsymbol{\mu}_i) - \boldsymbol{P}_i] r_i(\boldsymbol{z}) p_i(\boldsymbol{z}) \,\mathrm{d}\boldsymbol{z} - \boldsymbol{P}_i \right] \boldsymbol{P}_i^{-1} \boldsymbol{G}_i + O(s^2) \\ &= s \frac{1}{2} \boldsymbol{G}_i \boldsymbol{P}_i^{-1} \left[\int [(\boldsymbol{z} - \boldsymbol{\mu}_i) \circ (\boldsymbol{z} - \boldsymbol{\mu}_i) - \boldsymbol{P}_i] r_i(\boldsymbol{z}) p_i(\boldsymbol{z}) \,\mathrm{d}\boldsymbol{z} \right] \boldsymbol{P}_i^{-1} \boldsymbol{G}_i + O(s^2). \end{aligned} \tag{57}$$

Dividing by s and taking $s \to 0$ leads to the differential equation

$$\frac{\mathrm{d}\boldsymbol{G}_i}{\mathrm{d}t} = \frac{1}{2} \boldsymbol{G}_i \boldsymbol{P}_i^{-1} \left[\int [(\boldsymbol{z} - \boldsymbol{\mu}_i) \circ (\boldsymbol{z} - \boldsymbol{\mu}_i) - \boldsymbol{P}_i] r_i(\boldsymbol{z}) p_i(\boldsymbol{z}) \,\mathrm{d}\boldsymbol{z} \right] \boldsymbol{P}_i^{-1} \boldsymbol{G}_i. \tag{58}$$

This concludes the derivation for the governing equations of the population densities, trait means, and trait covariances of the species, given by Supplementary Eqs. 34, 46, and 58. Writing them out all together:

$$\frac{\mathrm{d}N_i}{\mathrm{d}t} = N_i \int r_i(\boldsymbol{z}) p_i(\boldsymbol{z}) \,\mathrm{d}\boldsymbol{z}, \tag{59}$$

$$\frac{\mathrm{d}\boldsymbol{\mu}_i}{\mathrm{d}t} = \boldsymbol{G}_i \boldsymbol{P}_i^{-1} \int (\boldsymbol{z} - \boldsymbol{\mu}_i) r_i(\boldsymbol{z}) p_i(\boldsymbol{z}) \,\mathrm{d}\boldsymbol{z}, \tag{60}$$

$$\frac{\mathrm{d}\boldsymbol{G}_i}{\mathrm{d}t} = \frac{1}{2} \boldsymbol{G}_i \boldsymbol{P}_i^{-1} \left[\int [(\boldsymbol{z} - \boldsymbol{\mu}_i) \circ (\boldsymbol{z} - \boldsymbol{\mu}_i) - \boldsymbol{P}_i] r_i(\boldsymbol{z}) p_i(\boldsymbol{z}) \,\mathrm{d}\boldsymbol{z} \right] \boldsymbol{P}_i^{-1} \boldsymbol{G}_i. \tag{61}$$

Supplementary Note 3 The consumer-resource model

Supplementary Eqs. 59, 60, and 61 only specify the dynamics if the per capita growth rates are given. Here we obtain these from a simple consumer-resource system. We have a set of resources which can

be mapped onto an L-dimensional trait space. For instance, if $L = 1$ and the trait is the bill depth of Darwin's finches, then the resources are of various sizes; the resource which a finch of bill depth $\boldsymbol{y}$ can optimally utilize is what we call a resource of size $\boldsymbol{y}$. Let $R(\boldsymbol{y})$ be the availability of resource $\boldsymbol{y}$. The degree to which an individual of phenotype $\boldsymbol{z}$ can utilize resource $\boldsymbol{y}$ is given by $u(\boldsymbol{z}, \boldsymbol{y})$. The amount of resource available is the maximum (saturation) concentration of the resource, $R_0(\boldsymbol{y})$, minus what has been consumed:

$$R(\boldsymbol{y}) = R_0(\boldsymbol{y}) - \sum_{j=1}^{S} \int u(\boldsymbol{z}', \boldsymbol{y}) N_j p_j(\boldsymbol{z}') \, \mathrm{d}\boldsymbol{z}', \tag{62}$$

where S is the number of consumer species, and $N_j p_j(\boldsymbol{z}')$ is the population density of consumer j's individuals that have phenotype $\boldsymbol{z}'$. In turn, the per capita growth rate $r(\boldsymbol{z})$ of consumers with phenotype $\boldsymbol{z}$ is proportional to their total resource consumption, and to a phenotype-specific mortality rate $m(\boldsymbol{z})$:

$$r(\boldsymbol{z}) = \int u(\boldsymbol{z}, \boldsymbol{y}) R(\boldsymbol{y}) \, \mathrm{d}y - m(\boldsymbol{z}). \tag{63}$$

The fact that $R(\boldsymbol{y})$ is directly expressed means the resources are assumed to operate on a fast time scale compared to the population dynamics [4], and are therefore always in a state of quasi-equilibrium. Also, resource depletion is weighted by the same function, $u(\boldsymbol{z}, \boldsymbol{y})$, as population growth in Supplementary Eq. 63. this means that the benefit an individual gains from resource $\boldsymbol{y}$ is proportional to its consumption of the same resource.

Substituting Supplementary Eq. 62 into Supplementary Eq. 63 yields

$$r(\boldsymbol{z}) = \int u(\boldsymbol{z}, \boldsymbol{y}) \left(R_0(\boldsymbol{y}) - \sum_{j=1}^{S} \int u(\boldsymbol{z}', \boldsymbol{y}) N_j p_j(\boldsymbol{z}') \, \mathrm{d}\boldsymbol{z}' \right) \mathrm{d}\boldsymbol{y} - m(\boldsymbol{z}). \tag{64}$$

Rearranging, we get

$$r(\boldsymbol{z}) = \underbrace{\left(\int u(\boldsymbol{z}, \boldsymbol{y}) R_0(\boldsymbol{y}) \, \mathrm{d}\boldsymbol{y} - m(\boldsymbol{z}) \right)}_{b(\boldsymbol{z})} - \sum_{j=1}^{S} \int \underbrace{\left(\int u(\boldsymbol{z}, \boldsymbol{y}) u(\boldsymbol{z}', \boldsymbol{y}) \, \mathrm{d}\boldsymbol{y} \right)}_{a(\boldsymbol{z}, \boldsymbol{z}')} N_j p_j(\boldsymbol{z}') \, \mathrm{d}\boldsymbol{z}'. \tag{65}$$

With the definitions

$$b(\boldsymbol{z}) = \int u(\boldsymbol{z}, \boldsymbol{y}) R_0(\boldsymbol{y}) \, \mathrm{d}\boldsymbol{y} - m(\boldsymbol{z}), \tag{66}$$

$$a(\boldsymbol{z}, \boldsymbol{z}') = \int u(\boldsymbol{z}, \boldsymbol{y}) u(\boldsymbol{z}', \boldsymbol{y}) \, \mathrm{d}\boldsymbol{y}, \tag{67}$$

this has the form of Lotka–Volterra growth with intrinsic rates $b(\boldsymbol{z})$ and competition kernel $a(\boldsymbol{z}, \boldsymbol{z}')$:

$$r(\boldsymbol{z}) = b(\boldsymbol{z}) - \sum_{j=1}^{S} N_j \int a(\boldsymbol{z}, \boldsymbol{z}') p_j(\boldsymbol{z}') \, \mathrm{d}\boldsymbol{z}'. \tag{68}$$

Assigning parameters to this model, the resource utilization curve $u(\boldsymbol{z}, \boldsymbol{y})$ is a Gaussian function of the difference between consumer phenotype $\boldsymbol{z}$ and resource quality $\boldsymbol{y}$. We write it as

$$u(\boldsymbol{z}, \boldsymbol{y}) = \left[(2\pi)^L \det(2\boldsymbol{W}) \right]^{1/4} \mathcal{N}(\boldsymbol{z}; \boldsymbol{y}, \boldsymbol{W}), \tag{69}$$

where $\mathcal{N}$ is given by Supplementary Eq. 1, and the factor in front is introduced for convenience, to simplify the expression for the competition kernel $a(\boldsymbol{z}, \boldsymbol{z}')$ between phenotypes $\boldsymbol{z}$ and $\boldsymbol{z}'$ in the future. The matrix $\boldsymbol{W}$ is the effective width of the utilization curve (the within-phenotype niche width of Taper & Case [2]), determining the range of resources that an individual with phenotype $\boldsymbol{z}$ can access.

The competition kernel $a(\boldsymbol{z}, \boldsymbol{z}')$ now reads, from Supplementary Eq. 67:

$$a(\boldsymbol{z}, \boldsymbol{z}') = \int u(\boldsymbol{z}, \boldsymbol{y}) u(\boldsymbol{z}', \boldsymbol{y}) \, \mathrm{d}\boldsymbol{y} = \left[(2\pi)^L \det(2\boldsymbol{W})\right]^{1/2} \int \mathcal{N}(\boldsymbol{z} - \boldsymbol{y}; \boldsymbol{0}, \boldsymbol{W}) \mathcal{N}(\boldsymbol{y}; \boldsymbol{z}', \boldsymbol{W}) \, \mathrm{d}\boldsymbol{y}. \quad (70)$$

The integral is the convolution of two normal functions, which is also normal with the summed means and covariances. We therefore have

$$a(\boldsymbol{z}, \boldsymbol{z}') = \sqrt{(2\pi)^L \det(2\boldsymbol{W})} \, \mathcal{N}(\boldsymbol{z}; \boldsymbol{z}', 2\boldsymbol{W}). \quad (71)$$

Introducing the notation $\boldsymbol{\Omega} = 2\boldsymbol{W}$ and using Supplementary Eq. 1, this simplifies to

$$a(\boldsymbol{z}, \boldsymbol{z}') = \exp\left(-\frac{1}{2}(\boldsymbol{z} - \boldsymbol{y})\boldsymbol{\Omega}^{-1}(\boldsymbol{z} - \boldsymbol{y})\right). \quad (72)$$

To determine $b(\boldsymbol{z})$ (Supplementary Eq. 66), we need to know the resource saturation densities $R_0(\boldsymbol{y})$ and the mortality rates $m(\boldsymbol{z})$. Generally, we are not concerned about their exact form, as long as they bound the region of growth for the species—either because $R_0(\boldsymbol{y})$ drops to zero at some point, or $m(\boldsymbol{z})$ becomes prohibitively large (or both). One possible parameterization proceeds by assuming that each resource saturates at the same level without consumption, and mortality increases with distance from a certain optimal point, which we take to be at $\boldsymbol{z} = \boldsymbol{0}$ without loss of generality. We thus define $R_0(\boldsymbol{y})$ as a constant, whose form is chosen for convenience to be

$$R_0(\boldsymbol{y}) = \text{const.} = \left[(2\pi)^L \det(\boldsymbol{\Omega})\right]^{-1/4}. \quad (73)$$

This way, the first term in $b(\boldsymbol{z})$ (Supplementary Eq. 66) is simply equal to 1:

$$\int u(\boldsymbol{z}, \boldsymbol{y}) R_0(\boldsymbol{y}) \, \mathrm{d}\boldsymbol{y} = \left[(2\pi)^L \det(\boldsymbol{\Omega})\right]^{-1/4} \int u(\boldsymbol{z}, \boldsymbol{y}) \, \mathrm{d}\boldsymbol{y}, \quad (74)$$

which, by Supplementary Eq. 69 and the fact that the normal distribution integrates to unity, is

$$\left[(2\pi)^L \det(\boldsymbol{\Omega})\right]^{-1/4} \int u(\boldsymbol{z}, \boldsymbol{y}) \, \mathrm{d}\boldsymbol{y} = \int \mathcal{N}(\boldsymbol{z}; \boldsymbol{y}, \boldsymbol{\Omega}/2) \, \mathrm{d}\boldsymbol{y} = 1. \quad (75)$$

The mortality $m(\boldsymbol{z})$ increases with increasing distance from $\boldsymbol{z} = \boldsymbol{0}$. We will use two possible forms: either a quadratically increasing mortality with $m(\boldsymbol{z}) = (\boldsymbol{z}\boldsymbol{z})/\theta^2$, or the quartic function $(\boldsymbol{z}) = (\boldsymbol{z}\boldsymbol{z})^2/\theta^4$. Here $(\boldsymbol{z}\boldsymbol{z})$ is the scalar product of $\boldsymbol{z}$ with itself, equal to the squared length of $\boldsymbol{z}$. Using Supplementary Eq. 75 and these expressions for the mortalities, we now obtain the intrinsic rates $b(\boldsymbol{z})$ from its definition in Supplementary Eq. 66:

$$b(z) = \int u(\boldsymbol{z}, \boldsymbol{y}) R_0(\boldsymbol{y}) \, \mathrm{d}\boldsymbol{y} - m(\boldsymbol{z}) = 1 - m(\boldsymbol{z}) = \begin{cases} 1 - \dfrac{(\boldsymbol{z}\boldsymbol{z})}{\theta^2} & \text{(quadratic mortality)} \\ 1 - \dfrac{(\boldsymbol{z}\boldsymbol{z})^2}{\theta^4} & \text{(quartic mortality)} \end{cases} \quad (76)$$

Without loss of generality, we fix $\theta = 1/2$. With this choice, positive growth can only be achieved in a sphere of diameter 1 in trait space. This means that all quantities with units of trait distance are measured relative to this diameter.

Supplementary Note 4 Eco-evolutionary dynamics in the consumer-resource model

We start from the per capita growth rates of Supplementary Eq. 68. Substituting this into our general eco-evolutionary dynamics (Supplementary Eqs. 59-61), we get

$$\frac{\mathrm{d}N_i}{\mathrm{d}t} = N_i \left[\int b(\boldsymbol{z}) p_i(\boldsymbol{z}) \, \mathrm{d}\boldsymbol{z} - \sum_{j=1}^{S} N_j \iint p_i(\boldsymbol{z}) a(\boldsymbol{z}, \boldsymbol{z}') p_j(\boldsymbol{z}') \, \mathrm{d}\boldsymbol{z}' \, \mathrm{d}\boldsymbol{z} \right], \quad (77)$$

$$\frac{\mathrm{d}\boldsymbol{\mu}_i}{\mathrm{d}t} = \boldsymbol{G}_i\boldsymbol{P}_i^{-1}\left[\int(\boldsymbol{z}-\boldsymbol{\mu}_i)b(\boldsymbol{z})p_i(\boldsymbol{z})\,\mathrm{d}\boldsymbol{z} - \sum_{j=1}^{S}N_j\iint(\boldsymbol{z}-\boldsymbol{\mu}_i)p_i(\boldsymbol{z})a(\boldsymbol{z},\boldsymbol{z}')p_j(\boldsymbol{z}')\,\mathrm{d}\boldsymbol{z}'\,\mathrm{d}\boldsymbol{z}\right], \tag{78}$$

$$\begin{aligned}\frac{\mathrm{d}\boldsymbol{G}_i}{\mathrm{d}t} = \frac{1}{2}\boldsymbol{G}_i\boldsymbol{P}_i^{-1}\Bigg[&\int[(\boldsymbol{z}-\boldsymbol{\mu}_i)\circ(\boldsymbol{z}-\boldsymbol{\mu}_i)-\boldsymbol{P}_i]b(\boldsymbol{z})p_i(\boldsymbol{z})\,\mathrm{d}\boldsymbol{z} \\ &-\sum_{j=1}^{S}N_j\iint[(\boldsymbol{z}-\boldsymbol{\mu}_i)\circ(\boldsymbol{z}-\boldsymbol{\mu}_i)-\boldsymbol{P}_i]p_i(\boldsymbol{z})a(\boldsymbol{z},\boldsymbol{z}')p_j(\boldsymbol{z}')\,\mathrm{d}\boldsymbol{z}'\,\mathrm{d}\boldsymbol{z}\Bigg]\boldsymbol{P}_i^{-1}\boldsymbol{G}_i.\end{aligned} \tag{79}$$

We introduce some simplifying notation:

$$b_i = \int b(\boldsymbol{z})p_i(\boldsymbol{z})\,\mathrm{d}\boldsymbol{z}, \tag{80}$$

$$\alpha_{ij} = \iint p_i(\boldsymbol{z})a(\boldsymbol{z},\boldsymbol{z}')p_j(\boldsymbol{z}')\,\mathrm{d}\boldsymbol{z}'\,\mathrm{d}\boldsymbol{z}, \tag{81}$$

$$\boldsymbol{g}_i = \int \boldsymbol{P}_i^{-1}(\boldsymbol{z}-\boldsymbol{\mu}_i)b(\boldsymbol{z})p_i(\boldsymbol{z})\,\mathrm{d}\boldsymbol{z}, \tag{82}$$

$$\boldsymbol{\beta}_{ij} = \iint \boldsymbol{P}_i^{-1}(\boldsymbol{z}-\boldsymbol{\mu}_i)p_i(\boldsymbol{z})a(\boldsymbol{z},\boldsymbol{z}')p_j(\boldsymbol{z}')\,\mathrm{d}\boldsymbol{z}'\,\mathrm{d}\boldsymbol{z}, \tag{83}$$

$$\boldsymbol{Q}_i = \int \boldsymbol{P}_i^{-1}[(\boldsymbol{z}-\boldsymbol{\mu}_i)\circ(\boldsymbol{z}-\boldsymbol{\mu}_i)-\boldsymbol{P}_i]\boldsymbol{P}_i^{-1}b(\boldsymbol{z})p_i(\boldsymbol{z})\,\mathrm{d}\boldsymbol{z}, \tag{84}$$

$$\boldsymbol{\Gamma}_{ij} = \iint \boldsymbol{P}_i^{-1}[(\boldsymbol{z}-\boldsymbol{\mu}_i)\circ(\boldsymbol{z}-\boldsymbol{\mu}_i)-\boldsymbol{P}_i]\boldsymbol{P}_i^{-1}p_i(\boldsymbol{z})a(\boldsymbol{z},\boldsymbol{z}')p_j(\boldsymbol{z}')\,\mathrm{d}\boldsymbol{z}'\,\mathrm{d}\boldsymbol{z}, \tag{85}$$

where $\boldsymbol{g}_i$ and $\boldsymbol{\beta}_{ij}$ are vectors, while $\boldsymbol{Q}_i$ and $\boldsymbol{\Gamma}_{ij}$ are matrices in trait space. With these notations, Supplementary Eqs. 77-79 are written as

$$\frac{\mathrm{d}N_i}{\mathrm{d}t} = N_i\left(b_i - \sum_{j=1}^{S}\alpha_{ij}N_j\right), \tag{86}$$

$$\frac{\mathrm{d}\boldsymbol{\mu}_i}{\mathrm{d}t} = \boldsymbol{G}_i\left(\boldsymbol{g}_i - \sum_{j=1}^{S}\boldsymbol{\beta}_{ij}N_j\right), \tag{87}$$

$$\frac{\mathrm{d}\boldsymbol{G}_i}{\mathrm{d}t} = \frac{1}{2}\boldsymbol{G}_i\left(\boldsymbol{Q}_i - \sum_{j=1}^{S}\boldsymbol{\Gamma}_{ij}N_j\right)\boldsymbol{G}_i. \tag{88}$$

To further relate Supplementary Eqs. 80-85 to one another, we calculate the first two derivatives of the normal distribution with respect to its mean. From Supplementary Eq. 1, the first derivative reads

$$\begin{aligned}\frac{\partial\mathcal{N}(\boldsymbol{z};\boldsymbol{\mu},\boldsymbol{P})}{\partial\boldsymbol{\mu}} = \frac{\partial\mathcal{N}(\boldsymbol{z};\boldsymbol{\mu},\boldsymbol{P})}{\partial\mu^k} &= -\frac{1}{2}\mathcal{N}(\boldsymbol{z};\boldsymbol{\mu},\boldsymbol{P})\frac{\partial}{\partial\mu^k}\sum_{m,n}(P^{-1})^{mn}(z^m-\mu^m)(z^n-\mu^n) \\ &= \frac{1}{2}\mathcal{N}(\boldsymbol{z};\boldsymbol{\mu},\boldsymbol{P})\sum_{m,n}(P^{-1})^{mn}(z^m\delta^{nk}+z^n\delta^{mk}-\delta^{mk}\mu^n-\delta^{nk}\mu^m) \\ &= \frac{1}{2}\mathcal{N}(\boldsymbol{z};\boldsymbol{\mu},\boldsymbol{P})\sum_{m,n}\left[(P^{-1})^{mk}z^m+(P^{-1})^{kn}z^n-(P^{-1})^{kn}\mu^n-(P^{-1})^{mk}\mu^m\right] \\ &= \mathcal{N}(\boldsymbol{z};\boldsymbol{\mu},\boldsymbol{P})\sum_{m}(P^{-1})^{km}(z^m-\mu^m) \\ &= \mathcal{N}(\boldsymbol{z};\boldsymbol{\mu},\boldsymbol{P})\boldsymbol{P}^{-1}(\boldsymbol{z}-\boldsymbol{\mu}),\end{aligned} \tag{89}$$

where μ^k is the kth trait-component of the vector $\boldsymbol{\mu}$, $(P^{-1})^{mn}$ is the (m,n)th entry of the matrix $\boldsymbol{P}^{-1}$, summations go from 1 to the number of trait dimensions L, and δ^{kl} is the Kronecker symbol (equal to 1 if $k=l$ and to 0 otherwise). Since $\mathcal{N}(\boldsymbol{z};\boldsymbol{\mu},\boldsymbol{P}) = \mathcal{N}(\boldsymbol{\mu};\boldsymbol{z},\boldsymbol{P})$ (Supplementary Eq. 1), the derivative with

respect to the independent variable (as opposed to the mean) immediately follows from Supplementary Eq. 89:

$$\frac{\partial \mathcal{N}(\boldsymbol{z};\boldsymbol{\mu},\boldsymbol{P})}{\partial \boldsymbol{z}} = \mathcal{N}(\boldsymbol{z};\boldsymbol{\mu},\boldsymbol{P})\boldsymbol{P}^{-1}(\boldsymbol{\mu}-\boldsymbol{z}). \tag{90}$$

Similarly, the second derivative with respect to the mean reads

$$\begin{aligned}
\frac{\partial^2 \mathcal{N}(\boldsymbol{z};\boldsymbol{\mu},\boldsymbol{P})}{\partial \boldsymbol{\mu}\circ\partial \boldsymbol{\mu}} &= \frac{\partial^2 \mathcal{N}(\boldsymbol{z};\boldsymbol{\mu},\boldsymbol{P})}{\partial \mu^k \partial \mu^l} = \frac{\partial}{\partial \mu^k}\left[\frac{\partial \mathcal{N}(\boldsymbol{z};\boldsymbol{\mu},\boldsymbol{P})}{\partial \mu^l}\right] \\
&= \frac{\partial}{\partial \mu^k}\left[\mathcal{N}(\boldsymbol{z};\boldsymbol{\mu},\boldsymbol{P})\sum_m (P^{-1})^{lm}(z^m-\mu^m)\right] \\
&= \frac{\partial \mathcal{N}(\boldsymbol{z};\boldsymbol{\mu},\boldsymbol{P})}{\partial \mu^k}\sum_m (P^{-1})^{lm}(z^m-\mu^m) + \mathcal{N}(\boldsymbol{z};\boldsymbol{\mu},\boldsymbol{P})\left[-\sum_m (P^{-1})^{lm}\delta^{mk}\right] \\
&= \mathcal{N}(\boldsymbol{z};\boldsymbol{\mu},\boldsymbol{P})\left[\sum_m (P^{-1})^{km}(z^m-\mu^m)\right]\left[\sum_m (P^{-1})^{lm}(z^m-\mu^m)\right] - \mathcal{N}(\boldsymbol{z};\boldsymbol{\mu},\boldsymbol{P})(P^{-1})^{lk} \\
&= \mathcal{N}(\boldsymbol{z};\boldsymbol{\mu},\boldsymbol{P})\Big[\big(\boldsymbol{P}^{-1}(\boldsymbol{z}-\boldsymbol{\mu})\big)\circ\big(\boldsymbol{P}^{-1}(\boldsymbol{z}-\boldsymbol{\mu})\big) - \boldsymbol{P}^{-1}\Big] \\
&= \mathcal{N}(\boldsymbol{z};\boldsymbol{\mu},\boldsymbol{P})\boldsymbol{P}^{-1}[(\boldsymbol{z}-\boldsymbol{\mu})\circ(\boldsymbol{z}-\boldsymbol{\mu}) - \boldsymbol{P}]\boldsymbol{P}^{-1},
\end{aligned} \tag{91}$$

where we used the fact that $\boldsymbol{P}$, and therefore its inverse, is symmetric. From $\mathcal{N}(\boldsymbol{z};\boldsymbol{\mu},\boldsymbol{P}) = \mathcal{N}(\boldsymbol{\mu};\boldsymbol{z},\boldsymbol{P})$ again, the second derivative with respect to the independent variable is the same,

$$\frac{\partial^2 \mathcal{N}(\boldsymbol{z};\boldsymbol{\mu},\boldsymbol{P})}{\partial \boldsymbol{z}\circ\partial \boldsymbol{z}} = \mathcal{N}(\boldsymbol{z};\boldsymbol{\mu},\boldsymbol{P})\boldsymbol{P}^{-1}[(\boldsymbol{z}-\boldsymbol{\mu})\circ(\boldsymbol{z}-\boldsymbol{\mu}) - \boldsymbol{P}]\boldsymbol{P}^{-1}. \tag{92}$$

With these results and the definitions in Supplementary Eqs. 80-85, we can relate $\boldsymbol{g}_i$, $\boldsymbol{\beta}_{ij}$, $\boldsymbol{Q}_i$, and $\boldsymbol{\Gamma}_{ij}$ to derivatives of b_i and α_{ij}, keeping in mind that $p_i(\boldsymbol{z})$ is given by Supplementary Eq. 24:

$$\frac{\partial b_i}{\partial \boldsymbol{\mu}_i} = \frac{\partial}{\partial \boldsymbol{\mu}_i}\int b(\boldsymbol{z})p_i(\boldsymbol{z})\,\mathrm{d}\boldsymbol{z} = \int b(\boldsymbol{z})\frac{\partial p_i(\boldsymbol{z})}{\partial \boldsymbol{\mu}_i}\,\mathrm{d}\boldsymbol{z} = \int \boldsymbol{P}_i^{-1}(\boldsymbol{z}-\boldsymbol{\mu}_i)b(\boldsymbol{z})p_i(\boldsymbol{z}) = \boldsymbol{g}_i, \tag{93}$$

$$\frac{\partial \alpha_{ij}}{\partial \boldsymbol{\mu}_i} = \iint \frac{\partial p_i(\boldsymbol{z})}{\partial \boldsymbol{\mu}_i}a(\boldsymbol{z},\boldsymbol{z}')p_j(\boldsymbol{z}')\,\mathrm{d}\boldsymbol{z}'\,\mathrm{d}\boldsymbol{z} = \iint \boldsymbol{P}_i^{-1}(\boldsymbol{z}-\boldsymbol{\mu}_i)p_i(\boldsymbol{z})a(\boldsymbol{z},\boldsymbol{z}')p_j(\boldsymbol{z}')\,\mathrm{d}\boldsymbol{z}'\,\mathrm{d}\boldsymbol{z} = \boldsymbol{\beta}_{ij}, \tag{94}$$

$$\begin{aligned}
\frac{\partial^2 b_i}{\partial \boldsymbol{\mu}_i\circ\partial \boldsymbol{\mu}_i} &= \frac{1}{2}\int b(\boldsymbol{z})\frac{\partial^2 p_i(\boldsymbol{z})}{\partial \boldsymbol{\mu}_i\circ\partial \boldsymbol{\mu}_i}\,\mathrm{d}\boldsymbol{z} \\
&= \frac{1}{2}\int b(\boldsymbol{z})p_i(\boldsymbol{z})\boldsymbol{P}_i^{-1}[(\boldsymbol{z}-\boldsymbol{\mu}_i)\circ(\boldsymbol{z}-\boldsymbol{\mu}_i) - \boldsymbol{P}_i]\boldsymbol{P}_i^{-1}\,\mathrm{d}\boldsymbol{z} = \boldsymbol{Q}_i,
\end{aligned} \tag{95}$$

$$\begin{aligned}
\frac{\partial^2 \alpha_{ij}}{\partial \boldsymbol{\mu}_i\circ\partial \boldsymbol{\mu}_i} &= \iint \frac{\partial p_i(\boldsymbol{z})}{\partial \boldsymbol{\mu}_i\circ\partial \boldsymbol{\mu}_i}a(\boldsymbol{z},\boldsymbol{z}')p_j(\boldsymbol{z}')\,\mathrm{d}\boldsymbol{z}\,\mathrm{d}\boldsymbol{z}' \\
&= \iint \boldsymbol{P}_i^{-1}[(\boldsymbol{z}-\boldsymbol{\mu}_i)\circ(\boldsymbol{z}-\boldsymbol{\mu}_i) - \boldsymbol{P}_i]\boldsymbol{P}_i^{-1}p_i(\boldsymbol{z})a(\boldsymbol{z},\boldsymbol{z}')p_i(\boldsymbol{z}')\,\mathrm{d}\boldsymbol{z}'\,\mathrm{d}\boldsymbol{z} = \boldsymbol{\Gamma}_{ij}.
\end{aligned} \tag{96}$$

Note that $\partial\alpha_{ij}/\partial\boldsymbol{\mu}_i$ is interpreted as $\partial_1\alpha(\boldsymbol{\mu}_i,\boldsymbol{\mu}_j)$, the derivative with respect to the first argument. This means that even when $\boldsymbol{\mu}_j = \boldsymbol{\mu}_i$, the derivative is taken only with respect to the first $\boldsymbol{\mu}_i$, treating $\boldsymbol{\mu}_j = \boldsymbol{\mu}_i$ formally distinct.

We can now determine the functions in Supplementary Eqs. 80-85 explicitly, based on the forms of $a(\boldsymbol{z},\boldsymbol{z}')$ (Supplementary Eq. 72) and $b(\boldsymbol{z})$ (Supplementary Eq. 76). We have, from the definition of α_{ij} in Supplementary Eq. 81,

$$\alpha_{ij} = \iint p_i(\boldsymbol{z})a(\boldsymbol{z},\boldsymbol{z}')p_j(\boldsymbol{z}')\,\mathrm{d}\boldsymbol{z}'\,\mathrm{d}\boldsymbol{z} = \int p_i(\boldsymbol{z})\left(\int a(\boldsymbol{z},\boldsymbol{z}')p_j(\boldsymbol{z}')\,\mathrm{d}\boldsymbol{z}'\right)\mathrm{d}\boldsymbol{z}. \tag{97}$$

The inner integral can be written as the convolution of two multinormal distributions:

$$\begin{aligned}\int a(z, z')p_j(z')\,\mathrm{d}z' &= \sqrt{(2\pi)^L \det(\boldsymbol{\Omega})} \int \mathcal{N}(z - z'; \mathbf{0}, \boldsymbol{\Omega})\, \mathcal{N}(z'; \boldsymbol{\mu}_j, \boldsymbol{P}_j)\,\mathrm{d}z' \\ &= \sqrt{(2\pi)^L \det(\boldsymbol{\Omega})}\, \mathcal{N}(z; \boldsymbol{\mu}_j, \boldsymbol{P}_j + \boldsymbol{\Omega}),\end{aligned} \tag{98}$$

where we used the fact that the convolution of two normal distributions is yet another normal distribution with the summed means and covariance matrices. The outer integral of Supplementary Eq. 97 is then also written as a convolution, yielding α_{ij}:

$$\begin{aligned}\alpha_{ij} &= \int p_i(z)\left(\int a(z, z')p_j(z')\,\mathrm{d}z'\right)\mathrm{d}z \\ &= \sqrt{(2\pi)^L \det(\boldsymbol{\Omega})} \int p_i(z)\mathcal{N}(z; \boldsymbol{\mu}_j, \boldsymbol{P}_j + \boldsymbol{\Omega})\,\mathrm{d}z \\ &= \sqrt{(2\pi)^L \det(\boldsymbol{\Omega})} \int \mathcal{N}(z; \boldsymbol{\mu}_i, \boldsymbol{P}_i)\, \mathcal{N}(z; \boldsymbol{\mu}_j, \boldsymbol{P}_j + \boldsymbol{\Omega})\,\mathrm{d}z \\ &= \sqrt{(2\pi)^L \det(\boldsymbol{\Omega})} \int \mathcal{N}(\boldsymbol{\mu}_i - z; \mathbf{0}, \boldsymbol{P}_i)\, \mathcal{N}(z; \boldsymbol{\mu}_j, \boldsymbol{P}_j + \boldsymbol{\Omega})\,\mathrm{d}z \\ &= \sqrt{(2\pi)^L \det(\boldsymbol{\Omega})}\, \mathcal{N}(\boldsymbol{\mu}_i; \boldsymbol{\mu}_j, \boldsymbol{P}_i + \boldsymbol{P}_j + \boldsymbol{\Omega}) \\ &= \sqrt{\frac{\det(\boldsymbol{\Omega})}{\det(\boldsymbol{P}_i + \boldsymbol{P}_j + \boldsymbol{\Omega})}} \exp\!\left(-\frac{1}{2}(\boldsymbol{\mu}_i - \boldsymbol{\mu}_j)(\boldsymbol{P}_i + \boldsymbol{P}_j + \boldsymbol{\Omega})^{-1}(\boldsymbol{\mu}_i - \boldsymbol{\mu}_j)\right).\end{aligned} \tag{99}$$

The total width of this function is $\boldsymbol{P}_i + \boldsymbol{P}_j + \boldsymbol{\Omega}$. Within a single species therefore ($i = j$), the total width is $2\boldsymbol{P} + \boldsymbol{\Omega}$. Since $\boldsymbol{W} = \boldsymbol{\Omega}/2$ (the within-phenotype niche width is half of $\boldsymbol{\Omega}$; Supplementary Note 3), the total width reads $2(\boldsymbol{P} + \boldsymbol{W})$. The covariance $2\boldsymbol{P}$ is also known as the between-phenotype niche width [2]. Therefore the total width of α_{ii} is the between-phenotype niche width plus twice the within-phenotype niche width.

Obtaining $\boldsymbol{\beta}_{ij}$ is simply a matter of using Supplementary Eq. 90 and $\boldsymbol{\beta}_{ij} = \partial\alpha_{ij}/\partial\boldsymbol{\mu}_i$ (Supplementary Eq. 94):

$$\boldsymbol{\beta}_{ij} = \frac{\partial\alpha_{ij}}{\partial\boldsymbol{\mu}_i} = \sqrt{(2\pi)^L \det(\boldsymbol{\Omega})}\frac{\partial}{\partial\boldsymbol{\mu}_i}\mathcal{N}(\boldsymbol{\mu}_i; \boldsymbol{\mu}_j, \boldsymbol{P}_i + \boldsymbol{P}_j + \boldsymbol{\Omega}) = (\boldsymbol{P}_i + \boldsymbol{P}_j + \boldsymbol{\Omega})^{-1}(\boldsymbol{\mu}_j - \boldsymbol{\mu}_i)\alpha_{ij}. \tag{100}$$

Similarly, we get $\boldsymbol{\Gamma}_{ij}$ from Supplementary Eq. 92 and $\boldsymbol{\Gamma}_{ij} = \partial^2\alpha_{ij}/(\partial\boldsymbol{\mu}_i \circ \partial\boldsymbol{\mu}_i)$ (Supplementary Eq. 96):

$$\begin{aligned}\boldsymbol{\Gamma}_{ij} = \frac{\partial^2\alpha_{ij}}{\partial\boldsymbol{\mu}_i \circ \partial\boldsymbol{\mu}_i} &= \sqrt{(2\pi)^L \det(\boldsymbol{\Omega})}\frac{\partial^2}{\partial\boldsymbol{\mu}_i \circ \partial\boldsymbol{\mu}_i}\mathcal{N}(\boldsymbol{\mu}_i; \boldsymbol{\mu}_j, \boldsymbol{P}_i + \boldsymbol{P}_j + \boldsymbol{\Omega}) \\ &= (\boldsymbol{P}_i + \boldsymbol{P}_j + \boldsymbol{\Omega})^{-1}\Big[(\boldsymbol{\mu}_i - \boldsymbol{\mu}_j) \circ (\boldsymbol{\mu}_i - \boldsymbol{\mu}_j) - (\boldsymbol{P}_i + \boldsymbol{P}_j + \boldsymbol{\Omega})\Big](\boldsymbol{P}_i + \boldsymbol{P}_j + \boldsymbol{\Omega})^{-1}\alpha_{ij}.\end{aligned} \tag{101}$$

We now turn to specifying b_i, $\boldsymbol{g}_i$, and $\boldsymbol{Q}_i$. They take on different forms depending on our choice of quadratic or quartic mortality functions (Supplementary Eq. 76). We consider each in turn.

- Quadratic function: $b(z) = 1 - (zz)/\theta^2$, where (zz) is again the scalar product of z with itself. We obtain b_i from Supplementary Eq. 80 via direct integration:

$$b_i = \int\left(1 - \frac{(zz)}{\theta^2}\right)p_i(z)\,\mathrm{d}z = 1 - \frac{\mathrm{trace}(\boldsymbol{P}_i) + (\boldsymbol{\mu}_i\boldsymbol{\mu}_i)}{\theta^2}. \tag{102}$$

From $\boldsymbol{g}_i = \partial b_i/\partial\boldsymbol{\mu}_i$ and $\boldsymbol{Q}_i = \partial^2 b_i/(\partial\boldsymbol{\mu}_i \circ \partial\boldsymbol{\mu}_i)$ (Supplementary Eqs. 93 and 95):

$$\boldsymbol{g}_i = \frac{\partial b_i}{\partial\boldsymbol{\mu}_i} = -\frac{2}{\theta^2}\boldsymbol{\mu}_i, \tag{103}$$

$$\boldsymbol{Q}_i = \frac{\partial^2 b_i}{\partial\boldsymbol{\mu}_i \circ \partial\boldsymbol{\mu}_i} = -\frac{2}{\theta^2}\boldsymbol{I}, \tag{104}$$

where $\boldsymbol{I}$ is the identity matrix.

- Quartic function: $b(\boldsymbol{z}) = 1 - (\boldsymbol{z}\boldsymbol{z})^2/\theta^4$. Again by direct integration,

$$\begin{aligned} b_i &= \int \left(1 - \frac{(\boldsymbol{z}\boldsymbol{z})^2}{\theta^4}\right) p_i(\boldsymbol{z})\,\mathrm{d}\boldsymbol{z} \\ &= 1 - \frac{\mathrm{trace}^2(\boldsymbol{P}_i) + 2\mathrm{trace}(\boldsymbol{P}_i^2) + 2\mathrm{trace}(\boldsymbol{P}_i)(\boldsymbol{\mu}_i\boldsymbol{\mu}_i) + 4\boldsymbol{\mu}_i\boldsymbol{P}_i\boldsymbol{\mu}_i + (\boldsymbol{\mu}_i\boldsymbol{\mu}_i)^2}{\theta^4}, \end{aligned} \tag{105}$$

and from $\boldsymbol{g}_i = \partial b_i/\partial\boldsymbol{\mu}_i$ and $\boldsymbol{Q}_i = \partial^2 b_i/(\partial\boldsymbol{\mu}_i \circ \partial\boldsymbol{\mu}_i)$,

$$\boldsymbol{g}_i = \frac{\partial b_i}{\partial\boldsymbol{\mu}_i} = -\frac{4}{\theta^4}[\mathrm{trace}(\boldsymbol{P}_i)\boldsymbol{\mu}_i + (\boldsymbol{\mu}_i\boldsymbol{\mu}_i)\boldsymbol{\mu}_i + 2\boldsymbol{P}_i\boldsymbol{\mu}_i], \tag{106}$$

$$\boldsymbol{Q}_i = \frac{\partial^2 b_i}{\partial\boldsymbol{\mu}_i \circ \partial\boldsymbol{\mu}_i} = -\frac{4}{\theta^4}[\mathrm{trace}(\boldsymbol{P}_i)\boldsymbol{I} + (\boldsymbol{\mu}_i\boldsymbol{\mu}_i)\boldsymbol{I} + 2(\boldsymbol{\mu}_i \circ \boldsymbol{\mu}_i) + 2\boldsymbol{P}_i]. \tag{107}$$

Supplementary Note 5 Calculating diversity

Supplementary Note 5.1 Discrete diversity

For a discrete number of categories C, there is a wide range of metrics for quantifying diversity. Here we use the Hill numbers of order q, denoted ${}^qD$. These are a class of metrics encompassing, e.g., the inverse Simpson and Shannon indices as special cases. Denoting the relative frequency of individuals in category i by f_i, these indices read

$${}^qD = \left(\sum_{i=1}^{C} f_i^q\right)^{\frac{1}{1-q}}. \tag{108}$$

The inverse Simpson index, which is the inverse of the probability that two randomly sampled individuals belong to the same category, is recovered for $q = 2$. While the expression is singular at $q = 1$, we can interpret ${}^1D$ as the limit of Supplementary Eq. 108 as q approaches 1, in which case it returns the exponential of the Shannon index: ${}^1D = \exp(-\sum_i f_i \log(f_i))$ [5].

If the categories are species, then $C = S$, and these indices return the usual measures of species diversity. For discrete functional traits, equating the categories with the possible trait states will yield the community's functional diversity. For instance, in a community of lepidopteran species where individuals have either white or melanistic black wing coloration, we have $C = 2$, f_1 is the frequency of white individuals (irrespective of species identity), and f_2 the frequency of melanistic ones.

Supplementary Note 5.2 Functional diversity in continuous trait spaces

For continuous traits (such as wing span, or the bill depth of darwin's finches, or the shell morphology of land snails), we are concerned with trait space coverage [6]. This means a measure of the fraction of trait space occupied by the community, and the evenness of this cover (Supplementary Figure 1). There are various ways of obtaining such a measure. Here we do so by using the trait probability density [7] of the community, and then applying standard diversity metrics to this distribution.

The community-wide trait probability density function, $\mathcal{D}(\boldsymbol{z})$, is the normalized sum of the trait distributions of all species:

$$\mathcal{D}(\boldsymbol{z}) = \frac{\sum_{i=1}^{S} N_i p_i(\boldsymbol{z})}{\int \sum_{k=1}^{S} N_k p_k(\boldsymbol{z})\,\mathrm{d}\boldsymbol{z}}. \tag{109}$$

The numerator is the sum of all one-species trait distributions (its shape given by $p_i(\boldsymbol{z})$ and the area under the curve by the population density N_i), while the denominator is a normalization factor, making sure that $\mathcal{D}(\boldsymbol{z})$ integrates to unity. The expression can be simplified:

$$\mathcal{D}(\boldsymbol{z}) = \frac{\sum_{i=1}^{S} N_i p_i(\boldsymbol{z})}{\sum_{k=1}^{S} N_k \underbrace{\int p_k(\boldsymbol{z})\,\mathrm{d}\boldsymbol{z}}_{1\ (\text{Eq. } 26)}} = \frac{\sum_{i=1}^{S} N_i p_i(\boldsymbol{z})}{\sum_{k=1}^{S} N_k} = \sum_{i=1}^{S} f_i p_i(\boldsymbol{z}), \tag{110}$$

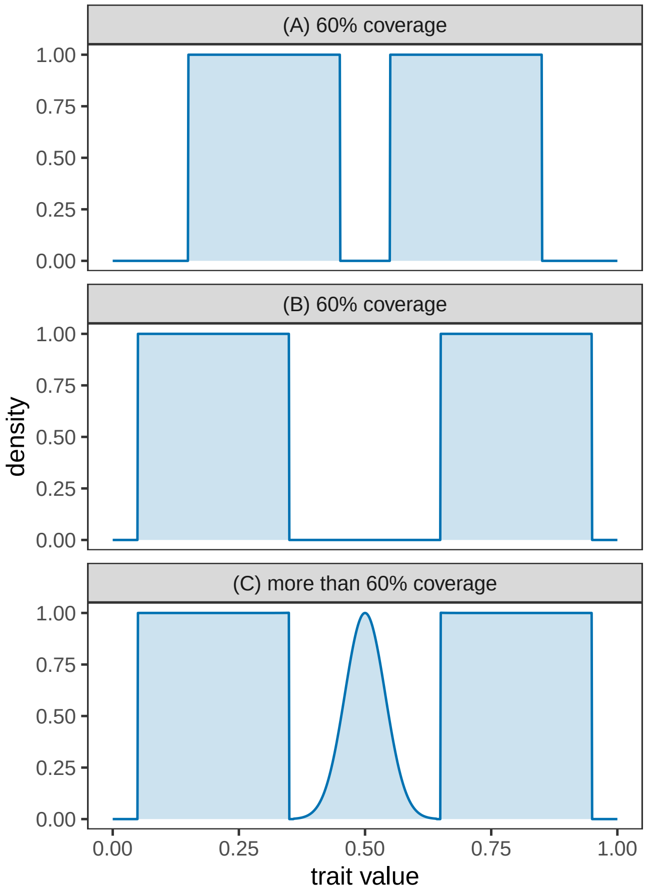

Supplementary Figure 1: Trait probability densities of three hypothetical communities, over a one-dimensional trait space where trait values may range from 0 to 1. (A): The trait probability density has two blocks in which trait values are uniformly distributed. Everywhere else, it is zero. Thus, in total, 60% of the trait axis is uniformly covered by the trait probability density function. (B): This community is as before, except there is a larger gap between the two regions of positive density. This, however, does not change the fact that still 60% of the trait space is covered, so this community is equally functionally diverse to the previous one. (C): As (B), but with an extra positive trait region in the middle. Although the total trait space coverage is 90% (all parts are covered except for the small 5% region at each end of the trait axis), coverage is not even. To determine the functional diversity of this community, a higher-order metric is needed.

where $f_i = N_i / \sum_{k=1}^{S} N_k$ is the relative frequency of species i in the community. It then appears sensible to define (differential) functional diversity measures by simply replacing f_i with $\mathcal{D}(z)$ and the sum with an integral in Supplementary Eq. 108. Unfortunately, doing so loses many good properties of ${}^qD$. Most problematically, it will no longer be invariant to changing the units of the traits in this naive continuum limit.

Instead, one should first create a finely subdivided grid of C cells, yielding a discretization of trait space. We then evaluate $\mathcal{D}(z)$ at the center of each cell, resulting in the value $\mathcal{D}_i$ in cell i. After normalizing the $\mathcal{D}_i$ with their sum to get $\hat{\mathcal{D}}_i = \mathcal{D}_i / \sum_k \mathcal{D}_k$, functional diversity can be obtained as before:

$$ {}^qD = \left(\sum_{i=1}^{C} \hat{\mathcal{D}}_i^q \right)^{\frac{1}{1-q}}. \tag{111} $$

To get an accurate diversity estimate, the trait space should be sufficiently finely discretized to make the difference between the $\mathcal{D}_i$ values in adjacent cells negligible—i.e., C should be large [8]. However, ${}^qD$ diverges as $C \to \infty$. For instance, if $\mathcal{D}(z)$ is a uniform distribution in the unit (hyper-)cube of dimension

L, then $\hat{\mathcal{D}}_i = 1/C$, and Supplementary Eq. 111 gives

$$ {}^qD = \left(\sum_{i=1}^{C} \left(\frac{1}{C} \right)^q \right)^{\frac{1}{1-q}} = \Big(C^{-q} \underbrace{\sum_{i=1}^{C} 1}_{C} \Big)^{\frac{1}{1-q}} = \left(C^{1-q} \right)^{\frac{1}{1-q}} = C \to \infty. \tag{112} $$

Fortunately, even though individual diversity values diverge as $C \to \infty$, the proportion of two such diversities does not. Therefore, the above metric works as long as we are interested in a relative measure of diversity across communities (which we are, in this work). One can even use this property to define a regularized measure of functional diversity, by dividing ${}^qD$ with the functional diversity of a community whose trait distribution is uniform in the same region of trait space as the original one—as seen from Supplementary Eq. 112, the functional diversity of such a community is always C. Redefining Supplementary Eq. 111 this way gives

$$ {}^qD = \frac{1}{C} \left(\sum_{i=1}^{C} \hat{\mathcal{D}}_i^q \right)^{\frac{1}{1-q}}, \tag{113} $$

which is a finite and well-defined measure of functional diversity even for an arbitrarily large C.

As an example, consider a community with a single trait ($L = 1$) that can take values between 0 and 1, and is uniform in the union of [0, 1/4] and [3/4, 1] (thus the middle half of the trait axis, [1/4, 3/4], is empty). Therefore, $\mathcal{D}(z) = 0$ for $1/4 \leq z \leq 3/4$ and 2 otherwise. Discretizing the [0, 1] interval into C equally spaced grid points, $\hat{\mathcal{D}}_i = 2/C$ for half the cells and $\hat{\mathcal{D}}_i = 0$ for the other (middle) half. Then, by Supplementary Eq. 113, we get

$$ {}^qD = \frac{1}{C} \Big(\underbrace{\sum_{i=1}^{C/2} \left(\frac{2}{C} \right)^q}_{(\frac{2}{C})^q \frac{C}{2}} + \underbrace{\sum_{i=\frac{C}{2}+1}^{C} 0^q}_{0} \Big)^{\frac{1}{1-q}} = \frac{1}{C} \left(\left(\frac{2}{C} \right)^q \frac{C}{2} \right)^{\frac{1}{1-q}} = \frac{1}{C} \left(\left(\frac{C}{2} \right)^{1-q} \right)^{\frac{1}{1-q}} = \frac{1}{2} \tag{114} $$

for any q, which is finite and meaningful even in the $C \to \infty$ limit.

Supplementary Eq. 113 can be expressed in an alternative but equivalent form, by using $\hat{\mathcal{D}}_i = \mathcal{D}_i / \sum_k \mathcal{D}_k$ and writing $\sum_k \mathcal{D}_k = C(C^{-1} \sum_k \mathcal{D}_k)$. The parenthesized quantity is the average of all $\mathcal{D}_k$; let us denote it by $\overline{\mathcal{D}}$. We then have

$$ {}^qD = \frac{1}{C} \left(\sum_{i=1}^{C} \left(\frac{\mathcal{D}_i}{C\overline{\mathcal{D}}} \right)^q \right)^{\frac{1}{1-q}} = \frac{1}{C} \left(C^{-q} \sum_{i=1}^{C} \frac{\mathcal{D}_i^q}{\overline{\mathcal{D}}^q} \right)^{\frac{1}{1-q}} = \left(\frac{1}{C} \sum_{i=1}^{C} \frac{\mathcal{D}_i^q}{\overline{\mathcal{D}}^q} \right)^{\frac{1}{1-q}}. \tag{115} $$

The expression in parentheses is the arithmetic average of $\mathcal{D}_i/\overline{\mathcal{D}}$. Denoting the vector of all $\mathcal{D}_i$ values simply by $\mathcal{D}$ and using overbars again to denote arithmetic averaging over each cell, functional diversity can be written in the simple form

$$ {}^qD = \left[\overline{\left(\mathcal{D}/\overline{\mathcal{D}} \right)^q} \right]^{\frac{1}{1-q}}. \tag{116} $$

In the main text, we always show results with $q = 2$, both for species diversity (Supplementary Eq. 108) and functional diversity (Supplementary Eq. 113). Here in the Supplementary Notes, we indicate the value of q in the figure captions.

Supplementary Note 5.3 Distance-dependent functional diversity metrics

The diversity metrics of Supplementary Eqs. 108 and 113 are distance-independent: only the extent and evenness of trait space coverage matter, not which parts of trait space are covered. However, there are diversity metrics which would score panel A of Supplementary Figure 1 as having lower diversity than

panel B, on the grounds that in B, the covered regions are further apart and therefore more varied. This approach is indispensable when one does not have access to trait probability densities in a continuous trait space. In that case, since the volume taken up by any number of discrete points in trait space is zero, the only way to account for the fact that a set of 100 points uniformly distributed in the [0, 1] range of a single trait is more diverse than another 100 distributed in [0.5, 0.6] (say) is to use a distance-sensitive metric. One such family of metrics is the diversity of order q [5], defined as

$$ {}^{q}D^{\boldsymbol{Z}} = \left(\sum_i f_i \left(\sum_j Z_{ij} f_j \right)^{q-1} \right)^{\frac{1}{1-q}} . \tag{117} $$

Here q is a sensitivity parameter (adjusting the relative weight assigned to common vs. rare types), f_i is the frequency of individuals having the ith trait value, and $\boldsymbol{Z}$ is a matrix with entries Z_{ij} that depends on the distance d_{ij} between the ith and jth trait values. One possible and common choice for Z_{ij} is

$$ Z_{ij} = \exp\left(-\frac{d_{ij}}{\xi} \right), \tag{118} $$

where ξ is a parameter controlling the distance beyond which two traits are sufficiently far apart to count as significantly different.

Since our approach uses trait probability densities and not discrete individuals, and we are interested in trait coverage in particular, it makes less sense to use distance-dependent metrics—from the point of view of the extent and evenness of the trait space covered, panels (A) and (B) of Supplementary Figure 1 should count as equally diverse. Unless there are independent reasons for believing that further-away regions covered by traits are more valuable for functional diversity, a distance-independent approach (assessing the fraction of trait space covered, and the evenness of this cover) is the correct one. Nevertheless, since distance-dependent metrics are important in other contexts, we did repeat our analyses measuring functional diversity using Supplementary Eq. 117 as well. Interestingly, switching to a distance-dependent metric did not change our basic results (Supplementary Note 8).

Supplementary Note 6 Species- and functional diversity in model communities

Supplementary Note 6.1 Simulation protocol

To explore the model's behavior, the following parameters were varied:

- Number of trait dimensions $L \in \{1, 2, 3\}$.
- Number of initial species $S \in \{2, 3, \ldots, 25\}$.
- Initial genetic covariances $\boldsymbol{G}_i(0) \in \{\text{low, high}\}$. They are generated via $\boldsymbol{G}_i(0) = \boldsymbol{U}_i \boldsymbol{B}_i \boldsymbol{U}_i^{\mathrm{T}}$, where $\boldsymbol{U}_i$ is a random orthogonal matrix and $\boldsymbol{B}_i$ is diagonal with nonzero entries sampled uniformly and independently from either [0.01, 0.05] (low) or [0.05, 0.1] (high).
- Environmental trait covariances $\boldsymbol{E}_i \in \{\text{low, medium, high}\}$. $\boldsymbol{E}_i$ is a diagonal matrix with nonzero entries sampled uniformly and independently from either [0.005, 0.008] (low), [0.015, 0.018] (medium), or [0.025, 0.028] (high).
- Competition width $\omega \in \{\text{low, high}\}$. We assume isotropic competition, meaning that the matrix $\boldsymbol{\Omega}$ (Supplementary Eq. 72) can be written in the form $\boldsymbol{\Omega} = (\omega^2/2)\boldsymbol{I}$, where ω^2 is the competition width in all directions in trait space, $\boldsymbol{I}$ is the identity matrix, and the factor $1/2$ is introduced for convenience. The low and high values of ω depend on trait dimension:
  - for $L = 1$, ω is either 0.1 (low) or 0.15 (high);

  - for $L = 2$, ω is either 0.25 (low) or 0.3 (high);
  - for $L = 3$, ω is either 0.4 (low) or 0.45 (high).

- Shape of intrinsic growth function $b(z) \in$ {quadratic, quartic} (Supplementary Eq. 76).

This leads to $3 \times 24 \times 2 \times 3 \times 2 \times 2 = 1728$ unique parameter combinations. Ten replicates were run for each parameterization. Each run was integrated for 10^{10} time units, to make sure the model communities reach an eco-evolutionarily stable equilibrium. Results are shown in Supplementary Figures 2-5.

A note about why environmental variances were very slightly randomized. In the model, groups of species will often undergo convergent evolution, evolving identical $\boldsymbol{\mu}_i$ and $\boldsymbol{G}_i$. Then, if all the $\boldsymbol{E}_i$ are equal as well, the species become indistinguishable, and therefore will coexist neutrally. However, this neutral coexistence is broken by the slightest difference in $\boldsymbol{E}_i$, with one of the species (possessing the "best" $\boldsymbol{E}_i$ value) driving all others in the group extinct. Since precisely equal $\boldsymbol{E}_i$ values across species are biologically implausible, this outcome would be artifactual. Slightly randomizing the $\boldsymbol{E}_i$ eliminates this artificial inflation of species diversity via neutrality.

Supplementary Note 6.2 More intrinsic growth shapes in one trait dimension

To further demonstrate that the results are insensitive to the shape of $b(z)$, we implemented three additional forms of this function:

- Rectangular function:

$$b(z) = \begin{cases} 1 & \text{if } -\theta \le z \le \theta \\ 0 & \text{otherwise} \end{cases} \tag{119}$$

- Triangular function:

$$b(z) = \begin{cases} \dfrac{z+\theta}{2\theta} & \text{if } -\theta \le z \le \theta \\ 0 & \text{otherwise} \end{cases} \tag{120}$$

- Bimodal function:

$$b(z) = 2(\theta^2 - z^2)\frac{(3+\sqrt{5})z^2 + 2\theta^2}{5\theta^4} \tag{121}$$

The value of θ, as before, is fixed at $\theta = 1/2$. The three functions are depicted in Supplementary Figure 6. Since these functions cannot be integrated analytically for more than one dimension in Supplementary Eq. 80, we restrict the analysis to single-dimensional trait spaces. Otherwise, we execute the same protocol as in Supplementary Note 6.1. The results (Supplementary Figures 7-8) are as before: functional diversity declines with species diversity.

Supplementary Note 6.3 Ecosystem functioning

One common measure of how well an ecosystem functions is the total community density (or biomass), $\sum_{i=1}^{S} N_i$. Another one is the number and quantity of resources utilized by the community as a whole. Here we show that, in our model, the two are identical up to a constant factor.

To do so, we obtain the expression for the total resource quantity utilized by the community, F. Since Supplementary Eq. 62 gives the available resource level as its maximum density $R_0(\boldsymbol{y})$ minus what has been consumed, $\sum_{j=1}^{S} \int u(\boldsymbol{z}', \boldsymbol{y}) N_j p_j(\boldsymbol{z}')\,\mathrm{d}\boldsymbol{z}'$, this second term, measures how much of resource $R(\boldsymbol{y})$ is being utilized. So the total resource use F is the integral of this quantity with respect to $\boldsymbol{y}$:

$$F = \int \left(\sum_{j=1}^{S} \int u(\boldsymbol{z}, \boldsymbol{y}) N_j p_j(\boldsymbol{z})\,\mathrm{d}\boldsymbol{z} \right) \mathrm{d}\boldsymbol{y}. \tag{122}$$

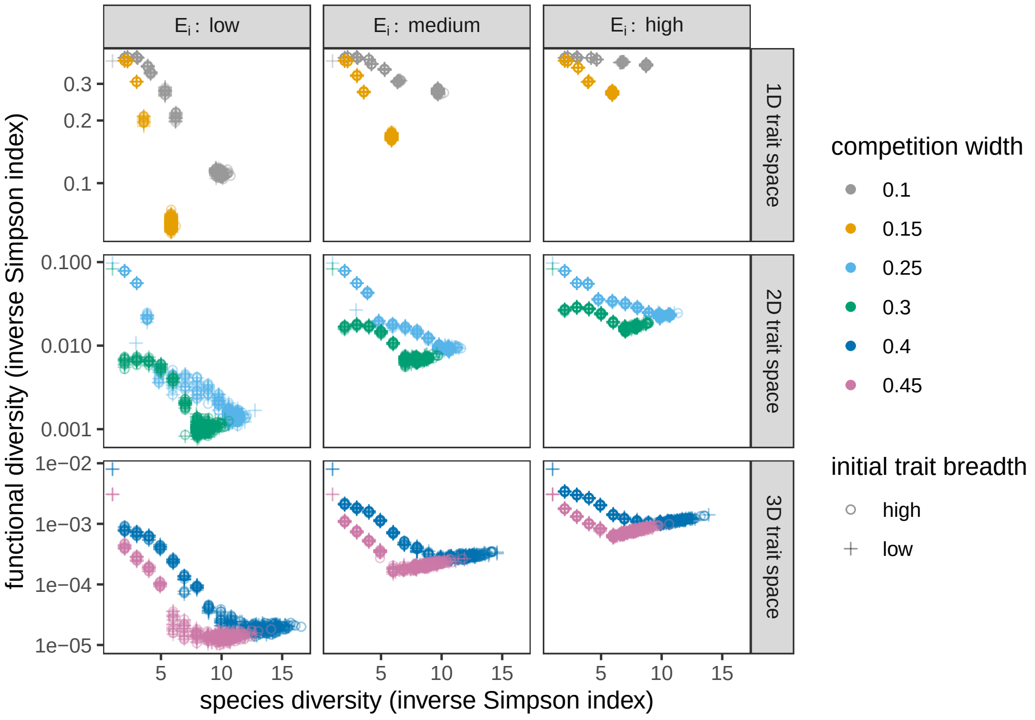

Supplementary Figure 2: Functional diversity against species diversity, for various dimensions L of trait space (rows) and levels of environmental trait variation (columns). Both diversities are measured via the inverse Simpson index ($q = 2$ in Supplementary Eqs. 108 and 113), with each trait axis discretized in the range $[-1, 1]$ into 101 bins for all calculations of functional diversity (Supplementary Note 5). This means that the number of bins is $C = 101^L$. Points show results across the parameter combinations and replicates; $n = 240$ independent samples per parameterization. Colors indicate different values of the competition width ω; shapes show results with high (○) and low (+) initial genetic variation. Intrinsic growth rates are quadratic (Supplementary Eq. 76) for all results shown.

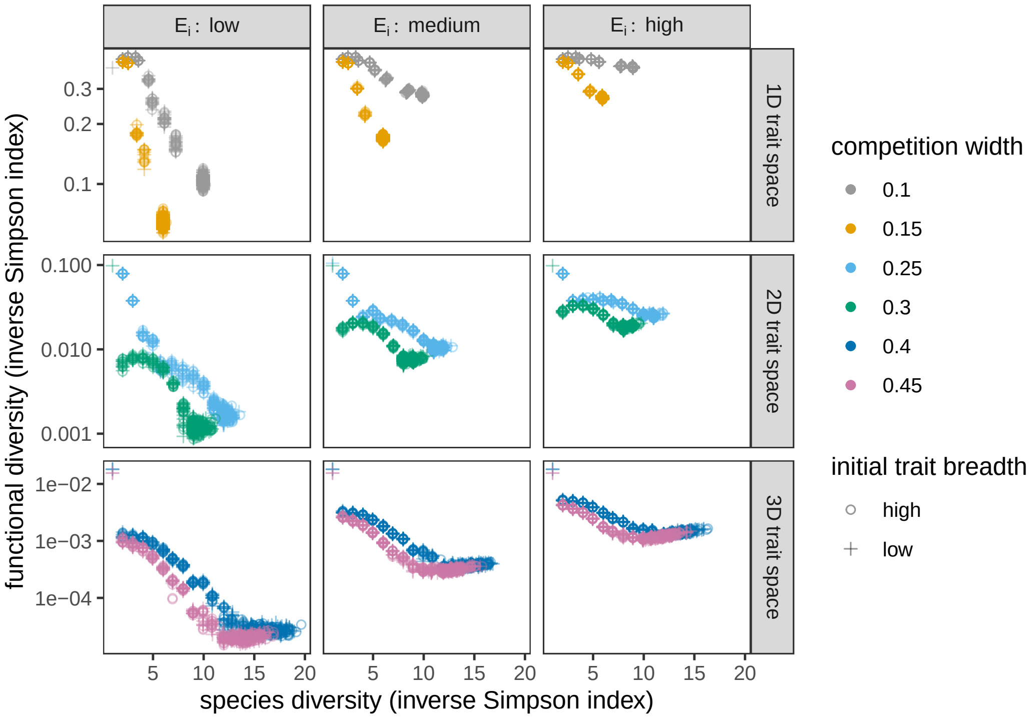

Supplementary Figure 3: As Supplementary Figure 2 ($n = 240$ independent samples per parameterization), with both species- and functional diversity measured by the inverse Simpson index ($q = 2$ in Supplementary Eqs. 108 and 113), and with quartic intrinsic growth rates (Supplementary Eq. 76).

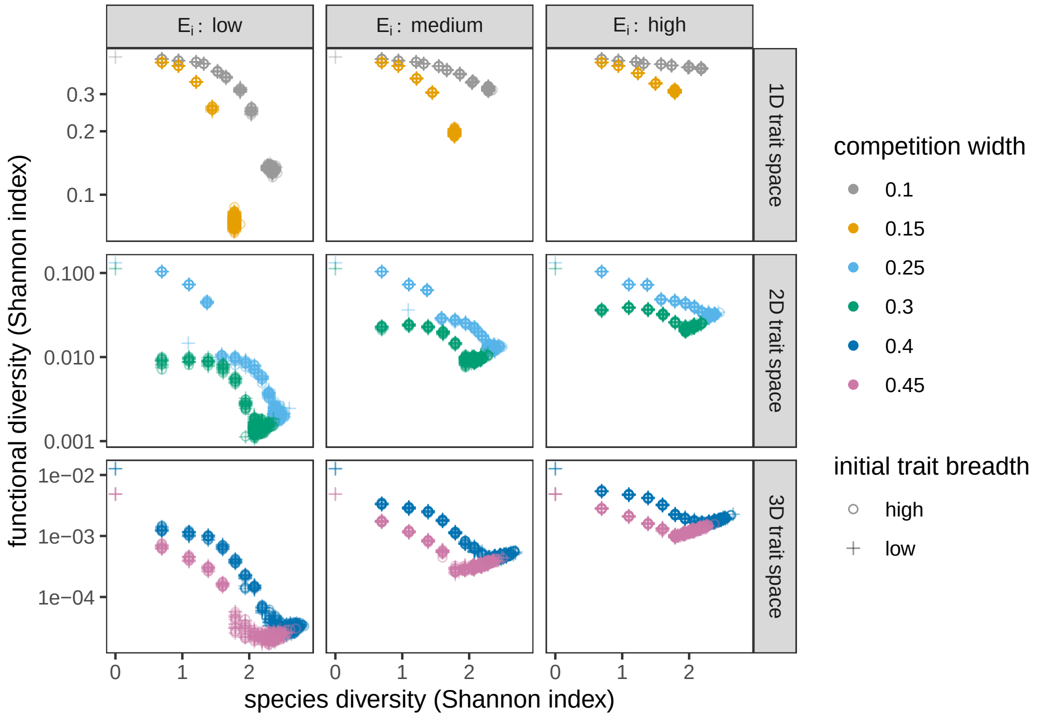

Supplementary Figure 4: As Supplementary Figure 2 (n = 240 independent samples per parameterization), with both species- and functional diversity measured by the Shannon index, and with quadratic intrinsic growth rates (Supplementary Eq. 76).

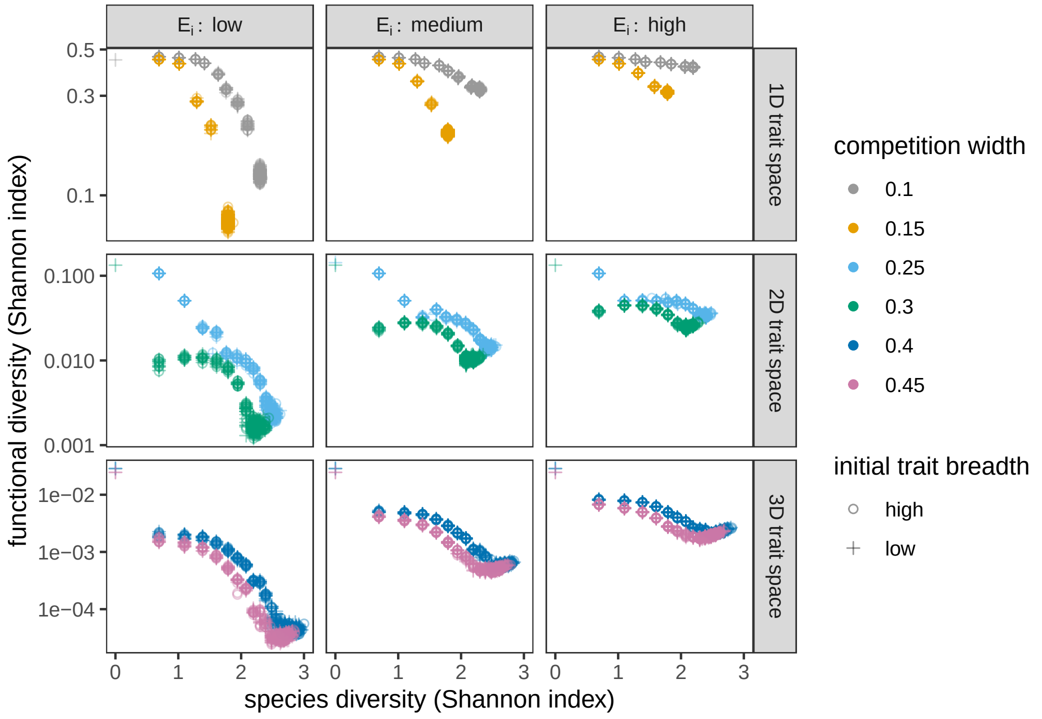

Supplementary Figure 5: As Supplementary Figure 2 (n = 240 independent samples per parameterization), with both species- and functional diversity measured by the Shannon index, and with quartic intrinsic growth rates (Supplementary Eq. 76).

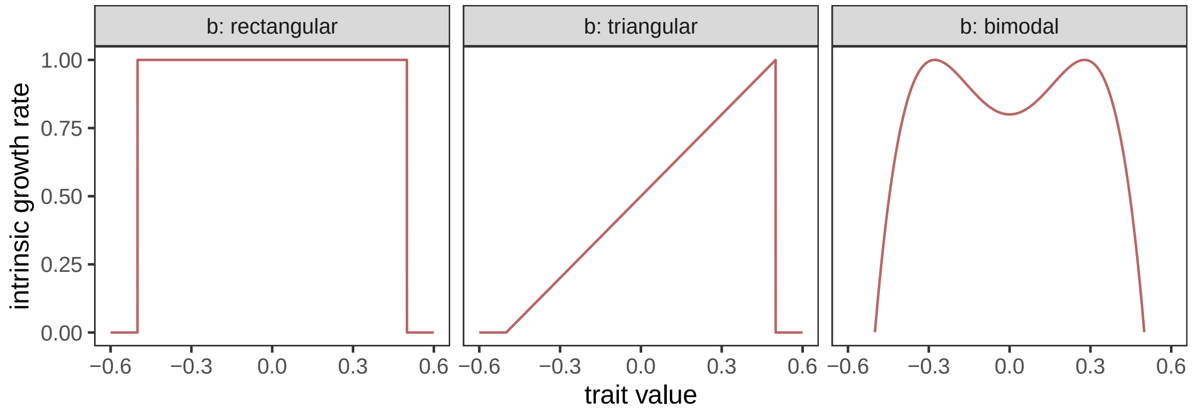

Supplementary Figure 6: Alternative shapes for the intrinsic growth function $b(z)$: rectangular (left; Supplementary Eq. 119), triangular (middle; Supplementary Eq. 120), and bimodal (right; Supplementary Eq. 121) forms.

Substituting $u(\boldsymbol{z}, \boldsymbol{y})$ from Supplementary Eq. 69 and $p_j(\boldsymbol{z})$ from Supplementary Eq. 24, and rearranging:

$$F = \sum_{j=1}^{S} N_j \left[(2\pi)^L \det(\boldsymbol{\Omega}) \right]^{1/4} \int \left(\int \mathcal{N}(\boldsymbol{z}; \boldsymbol{y}, \boldsymbol{\Omega}/2) \mathcal{N}(\boldsymbol{z}; \boldsymbol{\mu}_j, \boldsymbol{P}_j) \, \mathrm{d}\boldsymbol{z} \right) \mathrm{d}\boldsymbol{y}. \tag{123}$$

The parenthesized integral is the convolution of two normal functions, which is itself normal with the summed means and covariance matrices:

$$F = \sum_{j=1}^{S} N_j \left[(2\pi)^L \det(\boldsymbol{\Omega}) \right]^{1/4} \int \mathcal{N}(\boldsymbol{\mu}_j; \boldsymbol{y}, \boldsymbol{\Omega}/2 + \boldsymbol{P}_j) \, \mathrm{d}\boldsymbol{y}. \tag{124}$$

The integral over a normal distribution is 1, and the factor multiplying N_j is a constant that can be brought out before the summation. Therefore, we end up with

$$F = \left[(2\pi)^L \det(\boldsymbol{\Omega}) \right]^{1/4} \sum_{j=1}^{S} N_j, \tag{125}$$

which is indeed the total community density times a constant.

In the main text, we show results only with a quadratic growth function (Supplementary Eq 102) and for a limited set of competition widths. A more complete depiction of the results is in Supplementary Figure 9. As seen, ecosystem functioning still increases with species diversity, in all parameterizations. This is despite the fact that functional diversity itself declines with increasing species diversity.

Supplementary Note 6.4 Relaxing the assumption of Gaussian competition

Our model has a large array of ingredient functions and parameter settings which can be altered. To explore the model's behavior and its robustness against altering model assumptions, we have set up a fully-factorial numerical experimental protocol by systematically varying the number of trait dimensions, the initial number of species, the initial genetic covariance matrices, the environmental covariance matrices, the competition width, and the shape of the intrinsic growth function (Supplementary Note 6.1). These explorations revealed that our results are indeed highly robust.

That said, there are still assumptions left in the model that were never relaxed. For instance, the degree of competition between two phenotypes was always assumed to be a Gaussian function of the distance in phenotype space (Supplementary Eq. 72). This assumption was made because it allows one to analytically evaluate the integrals in the model's equations (Supplementary Note 4). We attempted to test

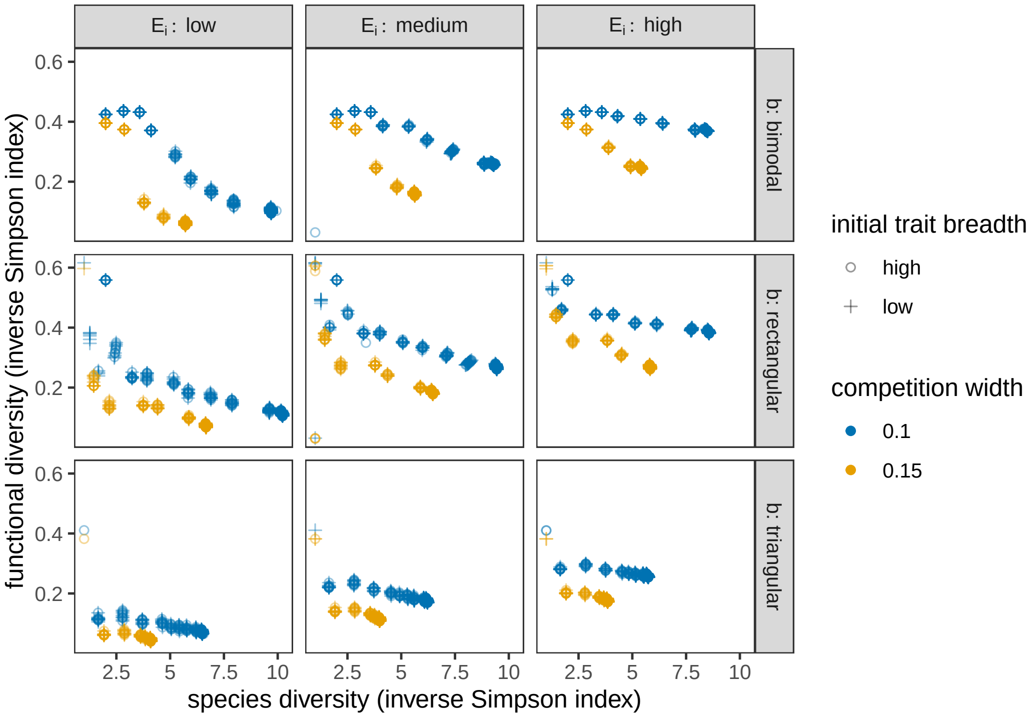

Supplementary Figure 7: Functional diversity against species diversity, in one-dimensional trait spaces, for various shapes of the intrinsic growth function $b(z)$ (rows) and levels of environmental trait variation (columns). Both diversities are measured via the inverse Simpson index ($q = 2$ in Supplementary Eqs. 108 and 113). Otherwise the figure is as Supplementary Figure 4 ($n = 240$ independent samples per parameterization).

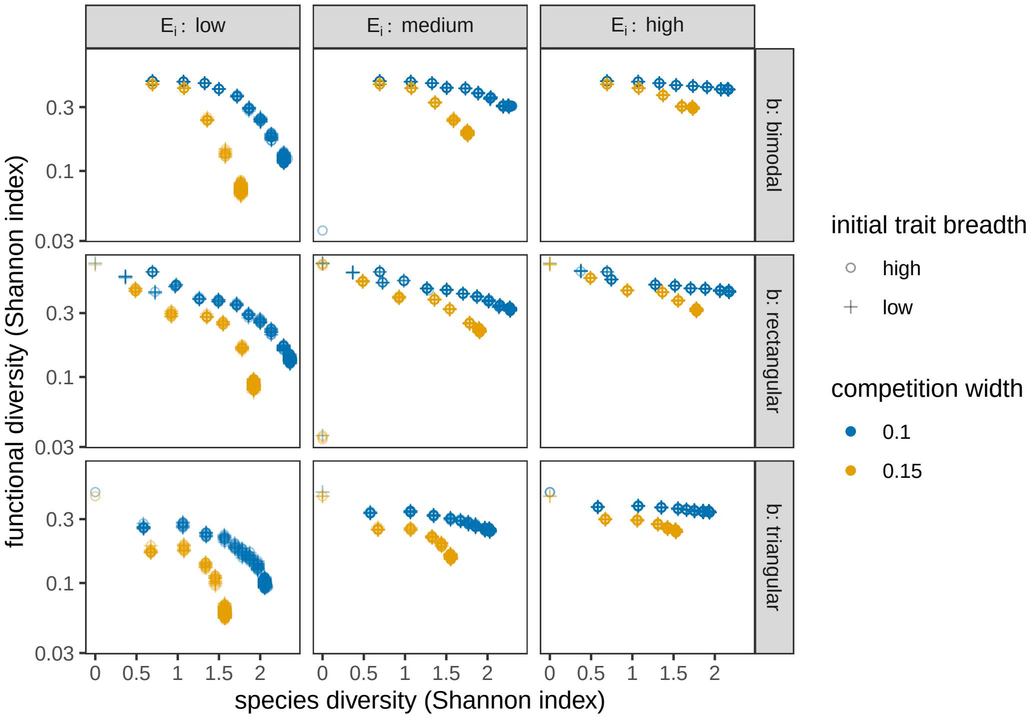

Supplementary Figure 8: As Supplementary Figure 7 ($n = 240$ independent samples per parameterization), but with both species- and functional diversity measured by the Shannon index.

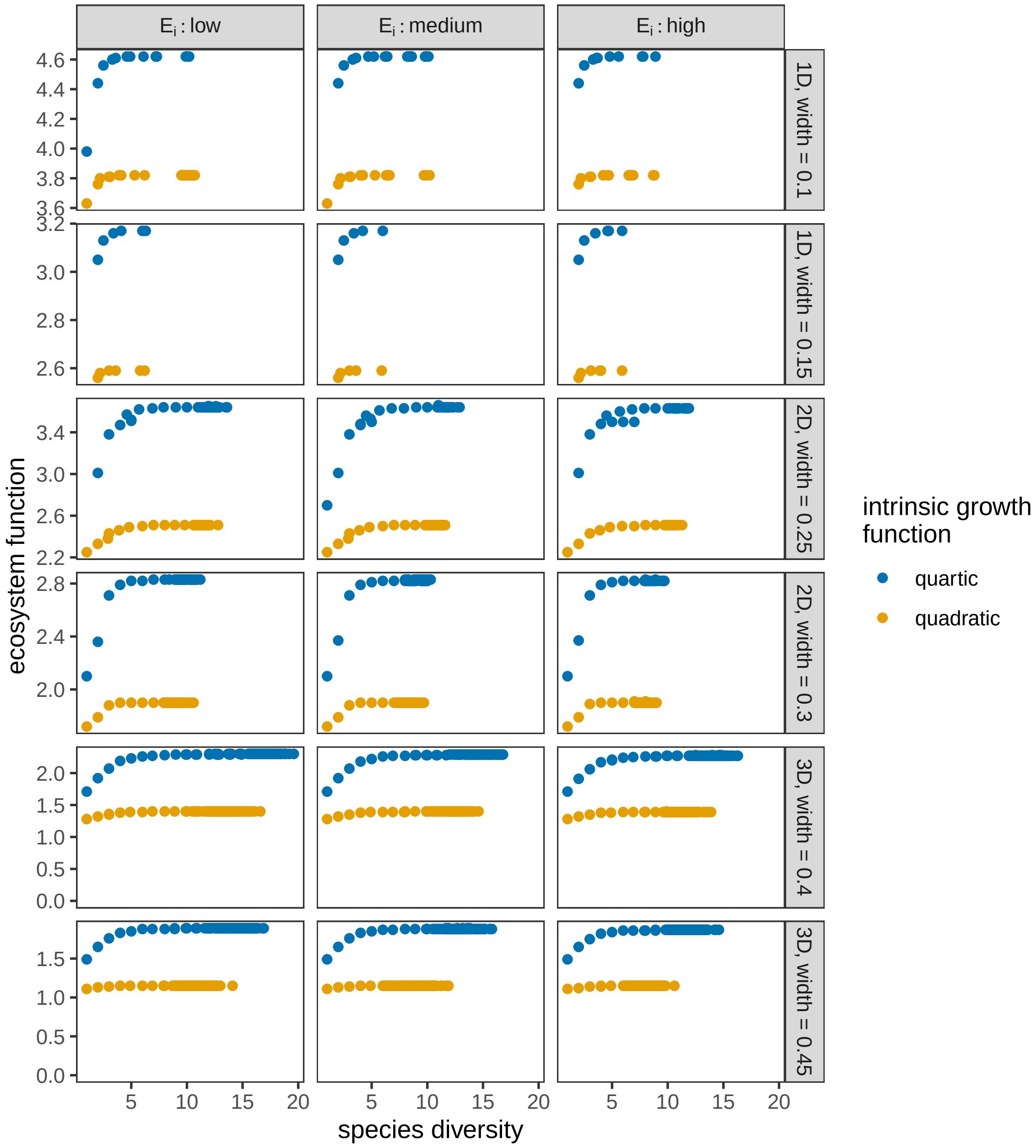

Supplementary Figure 9: Ecosystem functioning as a function of species diversity, for various values of trait space dimensionality and competition width (rows), levels of environmental trait variation (columns), and shapes of the intrinsic growth function (colors). There are $n = 480$ independent samples per parameterization. Ecosystem function is measured either as total community density or as the overall efficiency of resource utilization. Regardless of parameterization, ecosystem functioning always increases with increasing species diversity—even though functional diversity itself declines with increasing species diversity.

the limits of the model by trying to make assumptions which are biologically reasonable and yet overturn the negative relationship between species- and functional diversity. To this end, we implemented two new model variants. First, we replaced the Gaussian competition kernel with an asymmetric one. It is described by a scaled and shifted Gamma distribution, and it does not achieve its maximum at zero trait difference. Second, we abandoned the idea of localized competition (where phenotypes which are too far from one another do not interact appreciably) and implemented a hierarchical competition-mortality tradeoff model in the spirit of Adler & Mosquera [9]. These two competition kernels are depicted in Supplementary Figure 10.

Since these functions cannot be analytically jointly integrated with the trait distributions, one must resort to numerical integration to evaluate the right-hand sides of Supplementary Eqs. 59-61 governing the dynamics. This effectively restricts the analysis to one-dimensional trait spaces, because the computational

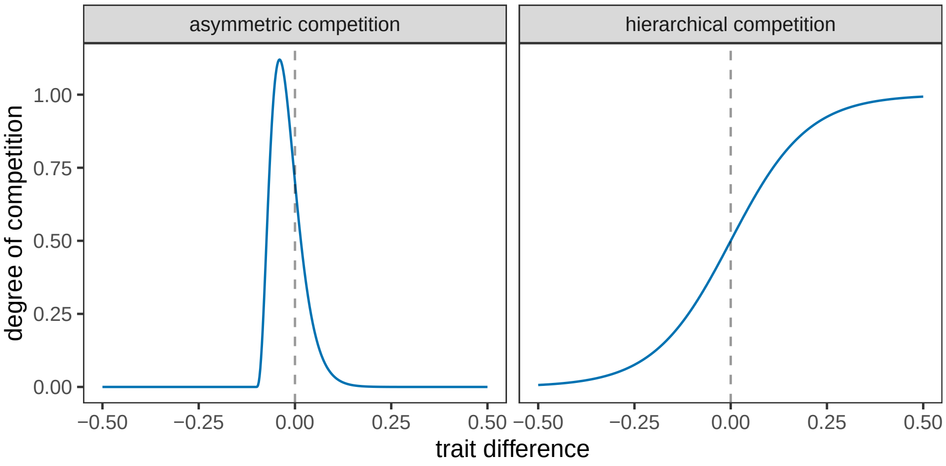

Supplementary Figure 10: Alternative forms for the competition kernel $a(z,z')$. Left: $a(z,z') = 10\Gamma((z-z')/\omega + 10, 4, 1/2)$, an asymmetric function where $\Gamma(z,\alpha,\beta)$ is the probability density function of the gamma distribution with shape parameter α and rate parameter β, and ω controls the width of the function (here, $\omega = 0.01$). Right: the hierarchical competition function $a(z,z') = (\tanh((z-z')/\omega) + 1)/2$ with a width of $\omega = 5$ (controlling how quickly the function transitions from low to high values). In this case, two phenotypes with substantially different trait values experience competition very differently: the phenotype with the lower trait value experiences no competition, whereas the one with the higher trait experiences strong competition. Biologically, this might correspond to competition for light between individuals of different heights, with height increasing in the negative direction along the trait axis.

effort increases exponentially with the number of dimensions over which one integrates. But for the one-dimensional case, we have modified and ran our model. The results are in Supplementary Figure 11.

As seen, having introduced some asymmetry into the competition and having made it peak away from zero trait difference did not generate any qualitative differences from our main results: there is still an overall negative relationship between species- and functional diversity. However, the relationship flips around and is positive for the case of hierarchical competition. This indicates the possible limits to our results, which may only hold under localized competition in trait space. Competition is "localized" if the competitive effect of two phenotypes on one another becomes negligible beyond some distance in trait space. Clearly, hierarchical competition is not localized: no matter how distant two phenotypes are, the competitive effect of the better competitor will remain substantial on the weaker one.

The reason for the inverted diversity pattern is also interesting, and is illustrated in Supplementary Figure 12. The lowest species in the hierarchy (i.e., the one with the largest mean trait value) always evolves a very broad trait distribution: this means that some small fraction of its individuals are actually very high up in the hierarchy. In fact, there is a more general positive relationship between trait mean and trait breadth: species that are on average lower in the hierarchy (higher trait values) have larger trait variance. This is sufficient to yield an overall positive relationship between species- and functional diversity.

In summary, our results are robust against altering practically every aspect of the model, except replacing localized competition with a hierarchical model. A detailed exploration of various non-localized modes of competition and their consequence for the relationship between species- and functional diversity is avenue for future research.

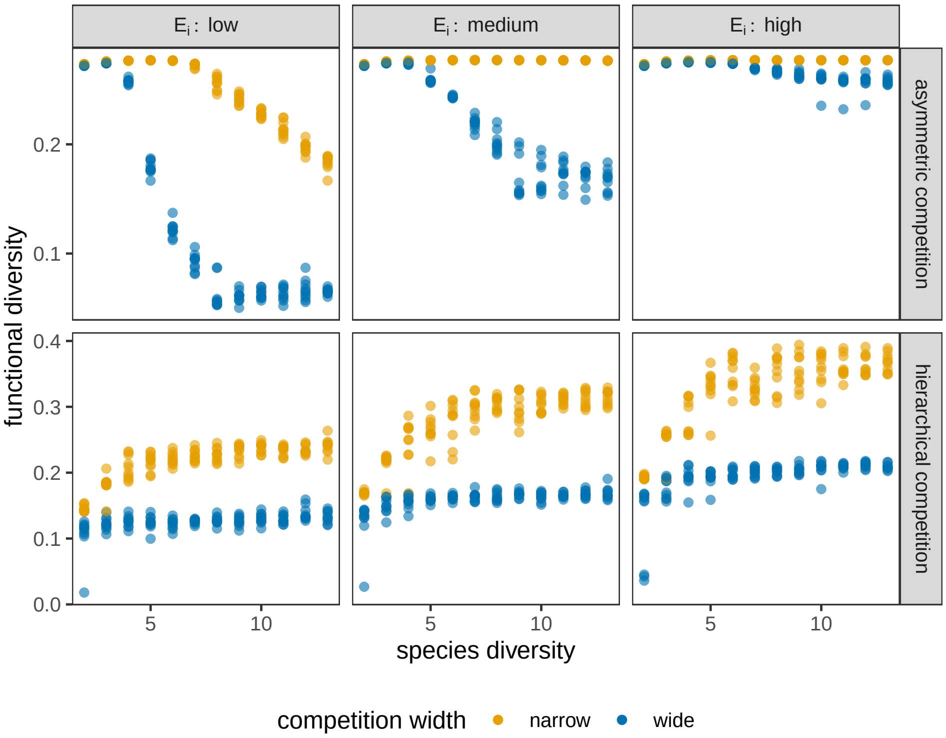

Supplementary Figure 11: Functional diversity against species diversity for the two competition kernel forms of Supplementary Figure 10 (rows). Colors represent smaller (yellow) and larger (blue) values of the competition width ω. There are $n = 120$ independent samples per parameterization. While the asymmetric case produces much the same pattern as before, the hierarchical model does not: in that case, functional diversity actually increases with species diversity. The intrinsic growth functions were either the quadratic $b(z) = 1 - 4z^2$ (asymmetric competition), or the monotonic $b(z) = 1 - \exp(z - 1/2)$ (hierarchical competition). This last choice means that the worse competitor a phenotype is in the hierarchy, the higher its growth rate, as per the standard assumptions of competition-mortality tradeoff models. However, there are diminishing returns beyond a point, because the function asymptotes at 1 for large values of z.

Supplementary Note 7 Species- and functional diversity in the empirical snail communities

Supplementary Note 7.1 General methodology

The empirical dataset is organized into a table where each row is a single individual, and columns record individuals' species identity, the community they belong to, and their morphological trait measurements. To obtain species diversity, we calculate the frequencies f_i in each community as the number of individuals of species i divided by the total number of individuals in that community. We then apply the diversity index of Supplementary Eq. 108, with C equal to the community-specific number of species. To obtain functional diversity, we first standardize every morphological variable by subtracting the mean and dividing the result by the standard deviation, calculated across the whole dataset. Then we decide the subset of morphological variables to be considered, making up the trait vector $\boldsymbol{z}$.

Next, we construct the community-wide trait probability density function $\mathcal{D}(\boldsymbol{z})$ from Supplementary Eq. 110, using the frequencies f_i and the fitted species-specific trait distributions $p_i(\boldsymbol{z})$. This fitting was done via one of two methods. First, multivariate normal distributions were fitted using the unbiased maximum likelihood estimators for the mean $\hat{\boldsymbol{\mu}}_i$ and covariance matrix $\hat{\boldsymbol{P}}_i$ of species i:

$$\hat{\boldsymbol{\mu}}_i = \frac{1}{N_i} \sum_{k=1}^{N_i} \boldsymbol{x}_{i,k} \tag{126}$$

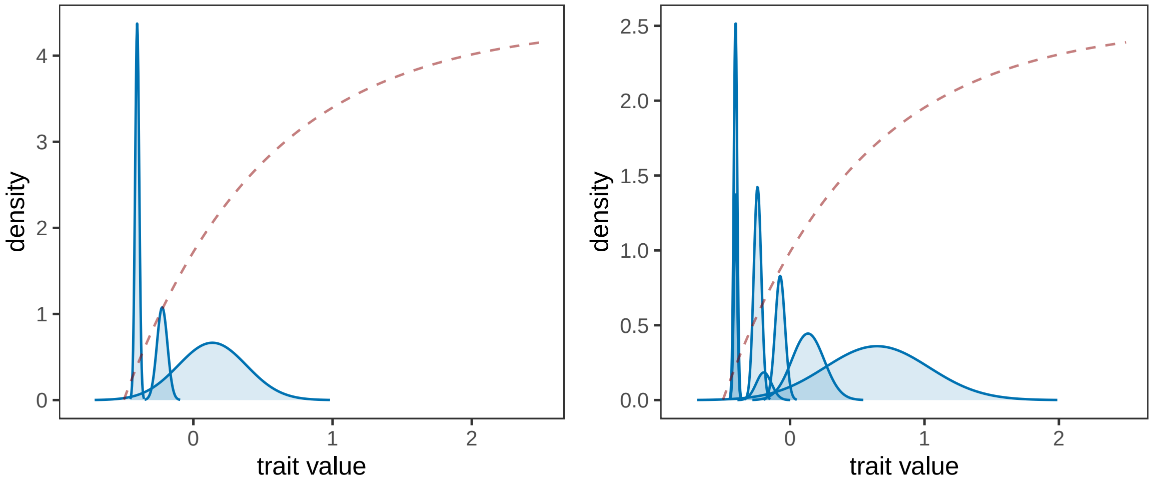

Supplementary Figure 12: Final steady state of a 3-species (left) and a 8-species (right) community, under hierarchical competition $a(z, z') = (\tanh((z - z')/5) + 1)/2$ and intrinsic growt $b(z) = 1 - \exp(z - 1/2)$ (red dashed curve). Species' trait distributions are shown by the filled curves, truncated at 99.9% of the total density; the area under the curve is the total population abundance. Unlike in the case of localized competition, only those species evolve narrow trait breadths whose mean trait values are low (meaning they are high up in the competitive hierarchy). The larger the trait means, the larger the trait variances get, which means that the original pattern of reduced functional diversity with increasing species diversity is overturned (see Supplementary Figure 11).

and

$$\hat{\boldsymbol{P}}_i = \frac{1}{N_i - 1} \sum_{k=1}^{N_i} (\boldsymbol{x}_{i,k} - \hat{\boldsymbol{\mu}}_i) \circ (\boldsymbol{x}_{i,k} - \hat{\boldsymbol{\mu}}_i), \tag{127}$$

where N_i is the number of individuals of species i in the given community, and $\boldsymbol{x}_{i,k}$ is the trait vector of species i's individual k. Second, instead of assuming a multinormal shape for the trait distributions, we also approximated them using multivariate kernel smoothing [7], obtaining a kernel density estimate from the data. This was done using the R package `ks` [10].

Once $\mathcal{D}(\boldsymbol{z})$ of the community is obtained from f_i, $p_i(\boldsymbol{z})$, and Supplementary Eq. 110, we apply Supplementary Eq. 113 for the functional diversity. Discretization is done within a (hyper-)cubic region of trait space which is the same for all communities (this makes them comparable), and which is large enough to encompass all trait distributions (with a minimal amount of truncation at the edges, since normal distributions technically reach out to infinity). The trait range is chosen broad enough so that any further increases to it no longer affect *relative* diversity values.[1] We then split this region into C equally-sized cubic bins, choosing C large enough to achieve convergence in Supplementary Eq. 113.

Supplementary Note 7.2 The Galápagos land snail data

Individuals of the land snail data belong to a particular island, and within the island, they either inhabit the arid or humid vegetation zone. All species belong in the genus *Naesiotus*. The distribution ranges of snail species never overlap across the arid and humid zones, so their species do not have the opportunity to interact with one another. This means that the species compositions of the humid and arid zones form effectively separate communities, and are treated here as such.

Three small satellite islands were removed from the data (CH, ED, and GA). In addition, there were only two sampled individuals of the species *Naesiotus achatellinus*; those were also removed (all other species had at least 13 individuals sampled, with most having at least 20 specimens). Snails were

[1] Evaluating functional diversity in ever-larger trait regions means that an ever-smaller fraction of that chosen region is actually filled up by trait values. This means that absolute functional diversity values decrease with increasing region size. However, we are only interested in comparing the relative diversity across communities, and these relative values are unaffected by increasing the trait volume.

placed and their functional diversity evaluated in a two-dimensional trait space whose axes correspond to centroid size and the first PC axis of shell shape [11]. This principal axis corresponds to whether shells are long and thin or compact and wide, and explains over 80% of all shell shape variation. The distribution of individuals in each subcommunity reveals that species segregate in this two-dimensional trait space, even though this segregation would not be evident by looking at only one of the two traits at a time (Supplementary Figure 13).

Raw species- and functional diversity data are shown in Supplementary Figure 14. This plot does not take into account the fact that islands vary greatly in their plant diversity. This is important because snail species partition the habitat according to the opportunities plants provide (leaves, trunks, logs, twigs, etc.), and it is thought that plant diversity is therefore tightly associated with habitat availability for snails. To correct for this, it is more reasonable to plot species diversity per host plant species in the given community. This is the figure shown in the main text.

The main text displays results assuming $q = 2$ in Supplementary Eq. 113. We also show that the obtained diversity patterns are robust to the choice of q (Supplementary Figure 15). Furthermore, when log-transforming species diversity per host plant species to create a more homogenously populated and normally distributed predictor range, a linear regression still yields an overall negative slope (Supplementary Figure 16).

There is one additional complication: the sampling methodology was such that relative abundances in the data do not express relative abundances on the islands. While knowing relative abundances is essential for diversity calculations, in our case the results are robust to simply randomizing abundances. We assigned a random abundance to each species, between 1 and 100, and recalculated both functional diversity and species diversity per host plant species. We then performed a linear regression and recorded whether its slope was negative. Repeating this procedure 1000 times, we found that the regression slope was negative in 911 cases. This reinforces the impression that there is no positive relationship, and even evidence of a negative one, between species- and functional diversity in this dataset.

These results were all obtained by fitting species' trait distributions with multivariate normal distributions. However, they are qualitatively unchanged if, instead of assuming that Gaussian distributions fit the data well, we instead approximate the trait distributions via kernel density estimation [7]. See Supplementary Figure 17 for the results.

Supplementary Note 8 Distance-dependent functional diversity metrics

Supplementary Figure 18 shows the same diversity information as Supplementary Figure 2, except with functional diversity measured by the distance-dependent metric of Supplementary Eq. 117. We set the sensitivity parameter to $q = 2$, in which case ${}^qD^{\mathbf{Z}}$ collapses to Rao's quadratic entropy measure [5]. Supplementary Figure 19 summarizes results for the empirical data. All results are qualitatively identical to those of the main text, for a wide range of distance parameters ξ. Switching to a distance-dependent functional diversity metric therefore does not affect our findings.

We reiterate however that these results are included for the purposes of illustration. Since our question concerns trait space coverage and its evenness, distance-independent metrics are a more natural choice in our case than the distance-dependent ones used in this section.

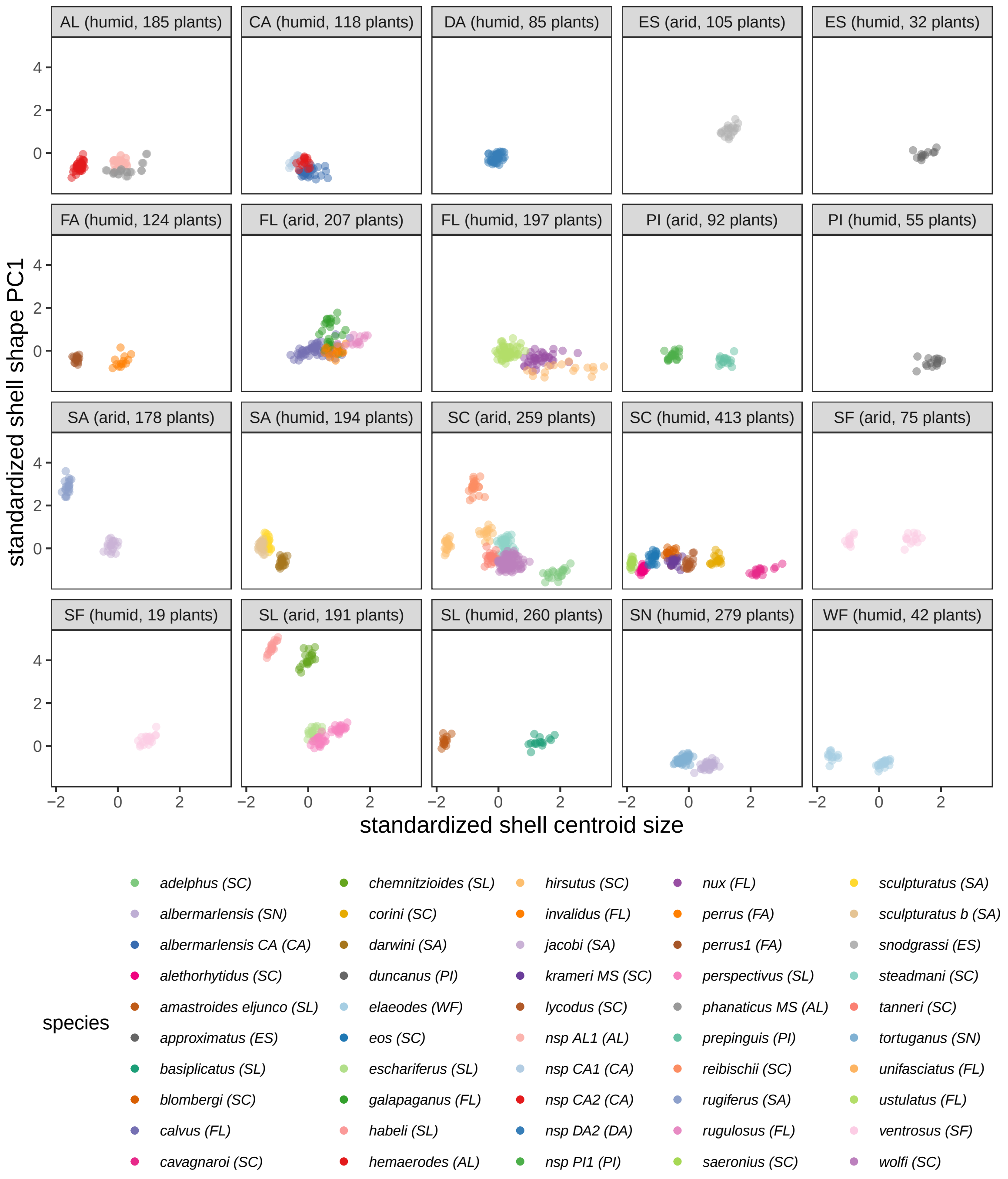

Supplementary Figure 13: Distribution of individuals in the two-dimensional trait space spanned by the standardized shell centroid size (abscissa) and standardized shell shape (ordinate; measured by the first PC axis of shape, explaining over 80% of shape variation). Each point is an individual belonging to a species (colors). Panels show independent subcommunities, indexed by island label and habitat type within the island (arid/humid). Island name abbreviations are: Alcedo Volcano (AL), Cerro Azul Volcano (CA), Darwin Volcano (DA), Espanola (ES), Fernandina (FA), Floreana (FL), Pinzon (PI), Santiago (SA), Santa Cruz (SC), Santa Fe (SF), San Cristobal (SL), Sierra Negra Volcano (SN), Wolf Volcano (WF). Panels also indicate the number of host plant species in each community, since the number of ecological opportunities are thought to scale with them [11]. Most species have published names, although a few are currently unpublished (e.g., "*corini*", "*krameri MS*", and "*phanaticus MS*"), or are morphospecies that are thought to be good biological species. Lineages corresponding to the type specimens are named normally (e.g., "*albemarlensis*"), while secondary lineages are named with an extra label (like "*albermarlensis CA*"). In this case, the label "*CA*" shows that the specimens are from a different volcano (Cerro Azul, in this case) than the type specimen. Another example is "*perrus1*", a population sampled from one side of Fernandina Island, while "*perrus*" (without any labels) is from the opposite side and represents a separate lineage.

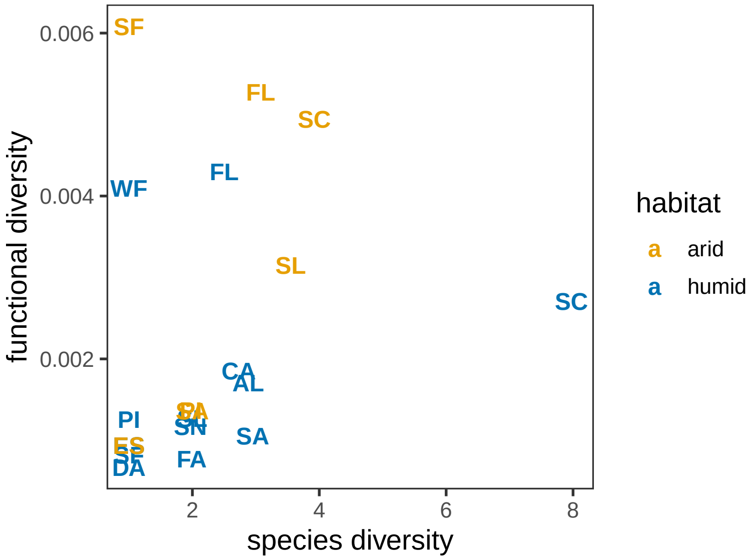

Supplementary Figure 14: Functional diversity against species diversity, measured by the inverse Simpson index (q = 2 in Supplementary Eqs. 108 and 113), for land snail communities on the Galápagos Islands. Species diversity is not corrected for the available host plant diversity in each community. Labels are island name abbreviations: Alcedo Volcano (AL), Cerro Azul Volcano (CA), Darwin Volcano (DA), Espanola (ES), Fernandina (FA), Floreana (FL), Pinzon (PI), Santiago (SA), Santa Cruz (SC), Santa Fe (SF), San Cristobal (SL), Sierra Negra Volcano (SN), Wolf Volcano (WF). Colors show communities in the arid (red) and humid (blue) zones of the islands, which form independent subcommunities (n = 20 independent samples).

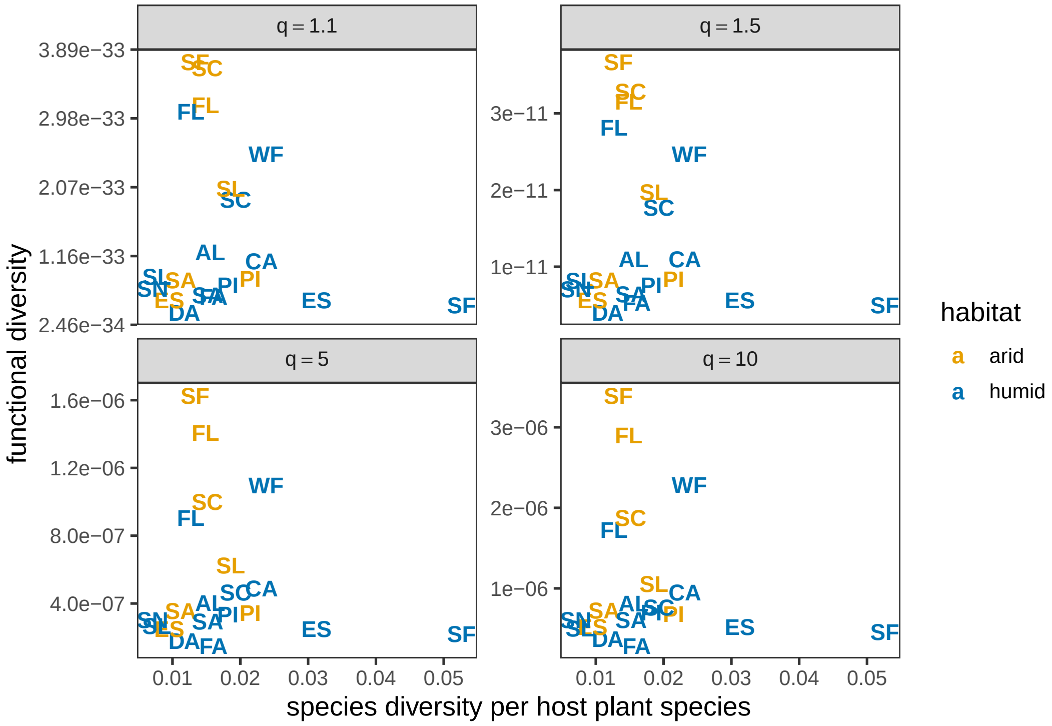

Supplementary Figure 15: Functional diversity against species diversity for land snail communities on the Galápagos Islands. Labels are island name abbreviations: Alcedo Volcano (AL), Cerro Azul Volcano (CA), Darwin Volcano (DA), Espanola (ES), Fernandina (FA), Floreana (FL), Pinzon (PI), Santiago (SA), Santa Cruz (SC), Santa Fe (SF), San Cristobal (SL), Sierra Negra Volcano (SN), Wolf Volcano (WF). Colors show communities in the arid (red) and humid (blue) zones of the islands, which form independent subcommunities (n = 20 independent samples in each panel). Species diversity is measured by the inverse Simpson index (q = 2 in Supplementary Eq. 108), while functional diversity is obtained from Supplementary Eq. 113 with various values of q (panels). As seen, results are robust to varying the sensitivity parameter q.

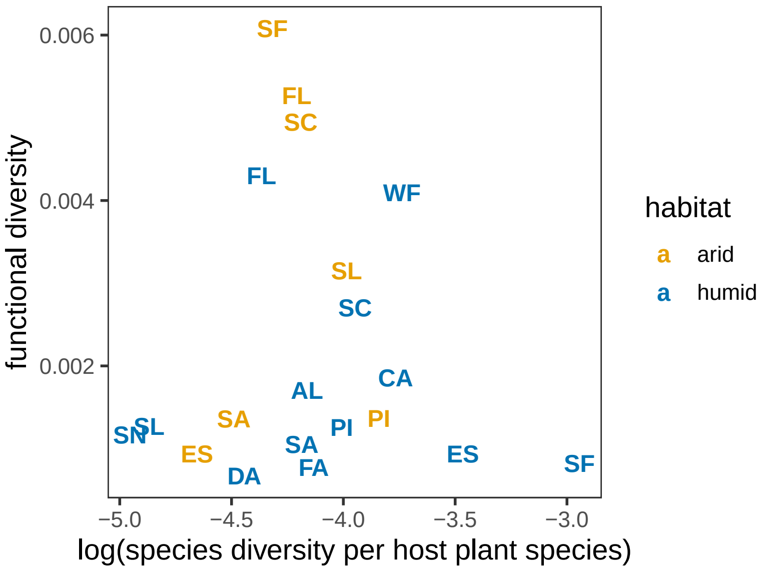

Supplementary Figure 16: As Supplementary Figure 14 (n = 20 independent samples), but q = 2 for both species- and functional diversity, species diversity is normalized by the number of plant species on the given island, and the abscissa is log-transformed. This creates a more homogenously populated and normally distributed range for the predictor: a Shapiro–Wilk test fails to reject normality (W = 0.97, p = 0.7; by contrast, normality is easily rejected when not taking logarithms: W = 0.78, p = 0.0004). Even after this transformation, there is no evidence for a positive relationship between the two diversity metrics. Though heteroscedasticity means that any linear regression should be treated as being merely illustrative, it yields a slope of $(-1.3 \pm 8.7) \times 10^{-4}$ with p = 0.88. [Further statistical information: Shapiro–Wilk and regression t-tests are two-sided; no correction for multiple comparison.]

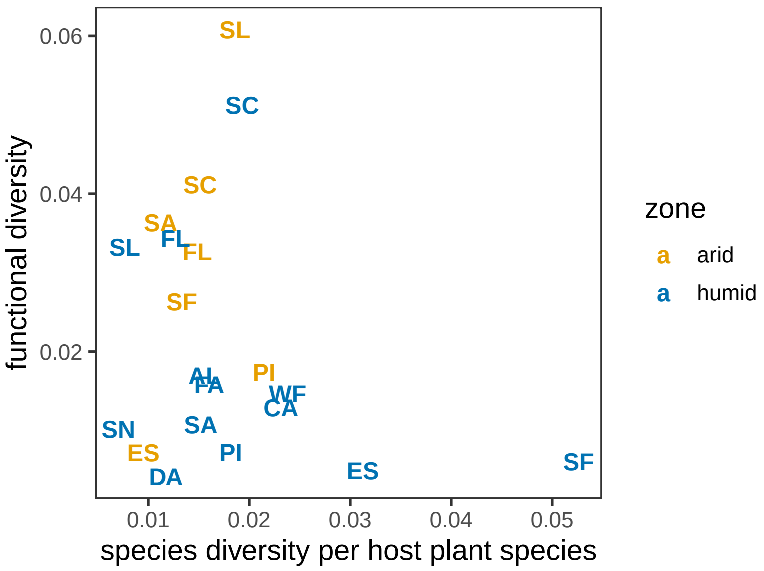

Supplementary Figure 17: As Supplementary Figure 14 (n = 20 independent samples), but with species diversity corrected for the number of available host plants (as in the main text), and species' trait distributions estimated via kernel smoothing instead of the fitting of bivariate normal distributions. Results are qualitatively unchanged: there is still a weak negative trend which is robust to randomizing relative abundances (out of 1000 randomizations, every single one ended up with a negative slope when regressing functional diversity against species diversity).

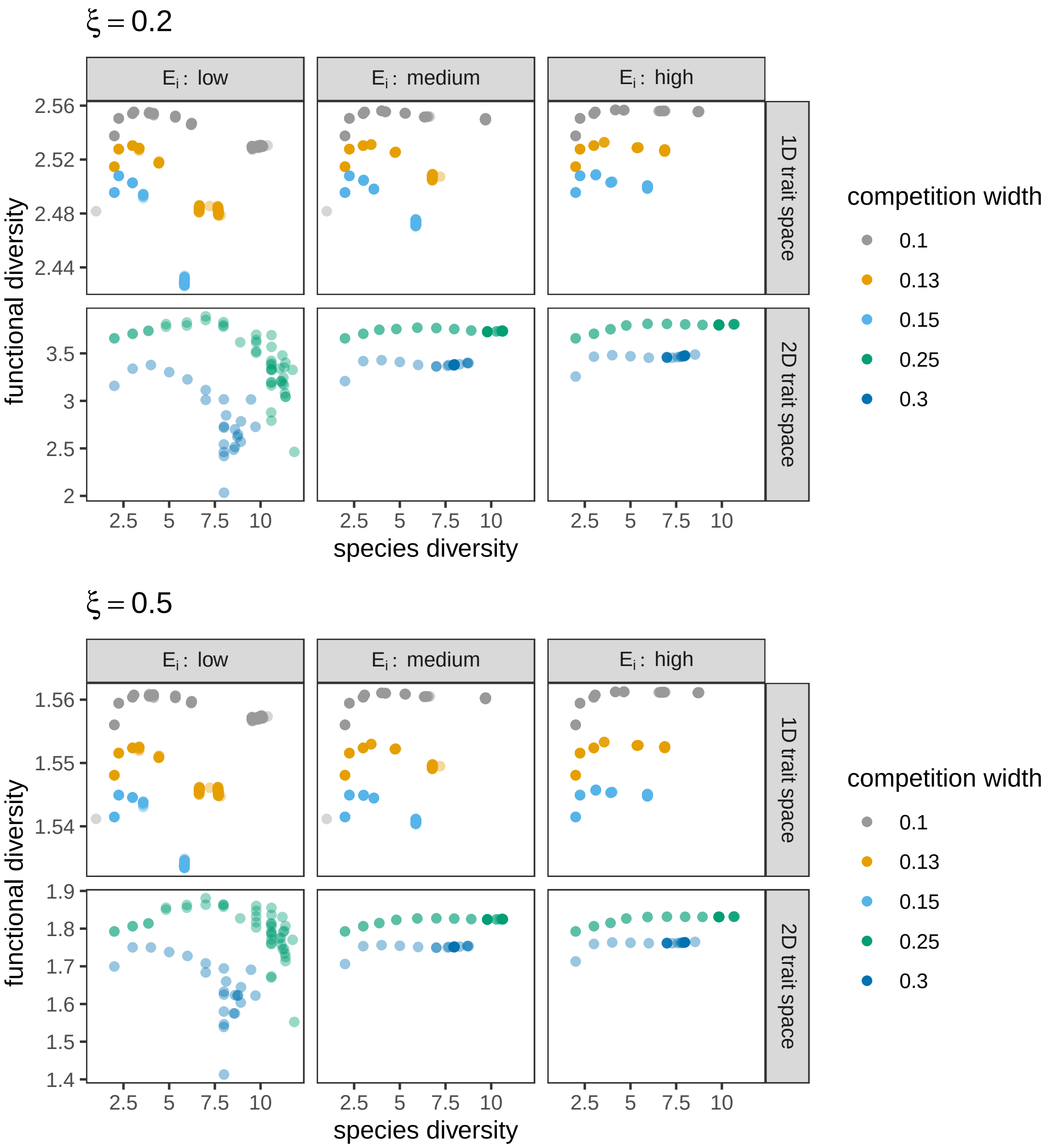

Supplementary Figure 18: Functional diversity (measured by the distance-dependent Supplementary Eq. 117 with $q = 2$) against species diversity (measured by Supplementary Eq. 108 with $q = 2$), in model communities, for distance parameter $\xi = 0.2$ (top panels) and $\xi = 0.5$ (bottom panels; the effective length of each trait dimension is 1 in the model). Results are qualitatively the same as with a distance-independent functional diversity metric (Supplementary Figure 2), but the increase of functional diversity at low species diversity values is more pronounced. Due to the computational intensity of obtaining this metric, we omitted three-dimensional trait spaces, and divided trait space into $C = 201$ equally-sized bins in the one-dimensional and $C = 67^2 = 4489$ ones in the two-dimensional case. There are $n = 240$ independent samples in all parameterizations with a one-dimensional trait space, and $n = 48$ independent samples in those with two trait dimensions.

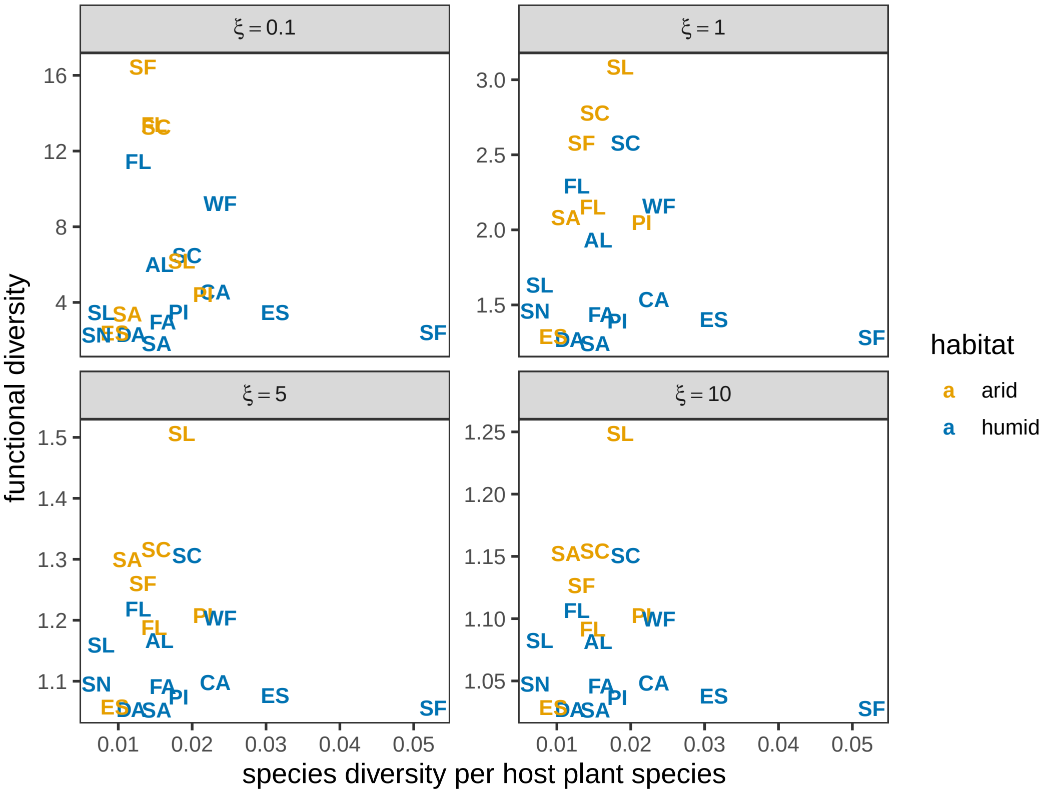

Supplementary Figure 19: Galápagos land snail functional diversity (measured by Supplementary Eq. 117 with $q = 2$; $n = 20$ independent samples per panel) against species diversity (Supplementary Eq. 108, $q = 2$). Panels show results for a wide range of ξ values. In all cases, the same qualitative results are retained as with the distance-independent functional diversity indices. Since the land snail dataset has the problem that the number of sampled individuals per species is not proportional to their relative abundances, we performed the same randomization procedure as in Supplementary Note 7.2 to make sure the results are robust to altering these relative abundances. Out of 100 randomizations, at least 90% had negative regression slopes for each of the four ξ values, indicating that the negative relationship between species- and functional diversity is robust.

Symbol	Description
S	Number of species
L	Dimensionality of trait space
$\boldsymbol{z}$	Vector in trait space
$\boldsymbol{m}$ / $\boldsymbol{f}$	Maternal / paternal genetic contribution to phenotype
$\boldsymbol{e}$	Environmental contribution to phenotype
$\boldsymbol{p}$	Phenotype vector of an individual; $\boldsymbol{p} = \boldsymbol{m} + \boldsymbol{f} + \boldsymbol{e}$
$\boldsymbol{G}$	Genetic covariance matrix; $\boldsymbol{G} = 2\mathrm{cov}(\boldsymbol{m}) = 2\mathrm{cov}(\boldsymbol{f})$
$\boldsymbol{E}$	Environmental covariance matrix; $\boldsymbol{E} = \mathrm{cov}(\boldsymbol{e})$
$\boldsymbol{P}$	Total phenotypic covariance matrix; $\boldsymbol{P} = \boldsymbol{G} + \boldsymbol{E}$
$\boldsymbol{p}_p$ / $\boldsymbol{p}_m$	Parent / Midparent phenotype vector
$\boldsymbol{\mu}$	Mean phenotype vector of species
$\mathcal{N}(\boldsymbol{z}; \boldsymbol{\mu}, \boldsymbol{P})$	Multivariate normal distribution with mean $\boldsymbol{\mu}$ and covariance matrix $\boldsymbol{P}$
$\boldsymbol{\mu}_{p_m,p}$	Mean of midparent-offspring regression
$\Sigma_{p_m,p}$	Covariance matrix of midparent-offspring regression
$p_i(z)$	Phenotype distribution of species i (multinormal with mean $\boldsymbol{\mu_i}$ and cov $\boldsymbol{P}_i$)
N_i	Population density of species i
$W_i(\boldsymbol{z})$	Discrete-time absolute fitness of species i's phenotype $\boldsymbol{z}$
$r_i(\boldsymbol{z})$	Continuous-time per capita growth rate species i's phenotype $\boldsymbol{z}$
$\boldsymbol{z} \circ \boldsymbol{\mu}$	Outer product of two vectors $\boldsymbol{z}$ and $\boldsymbol{\mu}$ (such that $[\boldsymbol{z} \circ \boldsymbol{\mu}]^{kl} = z^k \mu^l$)
$R(\boldsymbol{y})$	Resource density of type $\boldsymbol{y}$
$R_0(\boldsymbol{y})$	Saturation density of resource type $\boldsymbol{y}$
$m(\boldsymbol{z})$	Intrinsic mortality rate of phenotype $\boldsymbol{z}$
θ	Width of intrinsic mortality function; always set to $\theta = 1/2$
$u(\boldsymbol{z}, \boldsymbol{y})$	Utilization of resource $\boldsymbol{y}$ by a consumer of phenotype $\boldsymbol{z}$
$b(\boldsymbol{z})$	Effective intrinsic growth rate of phenotype $\boldsymbol{z}$
$a(\boldsymbol{z}, \boldsymbol{z}')$	Effective competition kernel between phenotypes $\boldsymbol{z}$ and $\boldsymbol{z}'$
$\boldsymbol{W}$	Breadth of resource utilization curve $u(\boldsymbol{z}, \boldsymbol{y})$ (within-phenotype niche width)
$\boldsymbol{\Omega}$	Breadth of effective competition kernel $a(\boldsymbol{z}, \boldsymbol{z}')$; $\boldsymbol{\Omega} = 2\boldsymbol{W}$
$\boldsymbol{I}$	Identity matrix
ω	Competition width, defining $\boldsymbol{\Omega} = (\omega^2/2)\boldsymbol{I}$
b_i	Species-level intrinsic growth rate, for species i
α_{ij}	Competition coefficient between species i and j
$\boldsymbol{B}$	Breadth of α_{ii} (between-phenotype niche width); $\boldsymbol{B} = 2(\boldsymbol{P} + \boldsymbol{W})$
$\boldsymbol{g}_i$	Selection pressure on species i's trait mean from growth
$\boldsymbol{\beta}_{ij}$	Selection pressure on species i's trait mean from competition with species j
$\boldsymbol{Q}_i$	Selection pressure on species i's trait covariance from growth
$\boldsymbol{\Gamma}_{ij}$	Selection pressure on species i's trait cov from competition with species j
$(\boldsymbol{z}\boldsymbol{z})$	Scalar product of $\boldsymbol{z}$ with itself (equal to squared length of $\boldsymbol{z}$)
$\mathrm{trace}(\boldsymbol{P})$	Trace (sum of diagonal entries) of the matrix $\boldsymbol{P}$
${}^qD$	Hill number (diversity index) of order q
${}^qD^{\boldsymbol{Z}}$	Distance-dependent diversity index of order q, with similarity matrix $\boldsymbol{Z}$
$\mathcal{D}(\boldsymbol{z})$	Trait probability density, as a function of trait value $\boldsymbol{z}$
C	Number of equally-sized grid cells to divide trait space into for diversity calculations
$\hat{\mathcal{D}}_i$	Normalized trait probability density in grid cell i
F	Total amount of resources utilized by the community
$\hat{\boldsymbol{\mu}}_i$	Maximum likelihood estimate of species i's trait mean
$\hat{\boldsymbol{P}}_i$	Maximum likelihood estimate of species i's trait covariance matrix

Supplementary Table 1: Table of symbols

Supplementary References

[1] Eaton, M. *Multivariate statistics: a vector space approach* (John Wiley and Sons, Ohio, US, 1983).

[2] Taper, M. L. & Case, T. J. Quantitative genetic models for the coevolution of character displacement. *Ecology* **66**, 355–371 (1985).

[3] Barabás, G. & D'Andrea, R. The effect of intraspecific variation and heritability on community pattern and robustness. *Ecology Letters* **19**, 977–986 (2016).

[4] MacArthur, R. H. Species packing and competitive equilibria for many species. *Theoretical Population Biology* **1**, 1–11 (1970).

[5] Leinster, T. & Cobbold, C. A. Measuring diversity: the importance of species similarity. *Ecology* **93**, 477–489 (2012).

[6] Fontana, S., Petchey, O. L. & Pomati, F. Individual-level trait diversity concepts and indices to comprehensively describe community change in multidimensional trait space. *Functional Ecology* **30**, 808–818 (2016).

[7] Carmona, C. P., de Bello, F., Mason, N. W. H. & Lepš, J. Traits without borders: integrating functional diversity across scales. *Trends in Ecology and Evolution* **31**, 382–394 (2016).

[8] Petchey, O. L. & Gaston, K. J. Functional diversity: back to basics and looking forward. *Ecology Letters* **9**, 741–758 (2006).

[9] Adler, F. R. & Mosquera, J. Is space necessary? Interference competition and limits to biodiversity. *Ecology* **81**, 3226–3232 (2000).

[10] R Core Team. *R: A Language and Environment for Statistical Computing*. R Foundation for Statistical Computing, Vienna, Austria (2021). URL `https://www.R-project.org/`.

[11] Parent, C. E. & Crespi, B. J. Ecological opportunity in adaptive radiation of Galápagos endemic land snails. *American Naturalist* **174**, 898–905 (2009).
