# Supplementary material for: The evolution of trait variance creates a tension between species diversity and functional diversity
Source: Nat Commun. 2022 May 9;13:2521. doi: 10.1038/s41467-022-30090-4 (PMC9085882; doi:10.1038/s41467-022-30090-4)
Supplement: Supplementary file 3 — Reporting Summary [file 41467_2022_30090_MOESM3_ESM.pdf]

nature portfolio

Corresponding author(s): György Barabás

Last updated by author(s): Mar 14, 2022

Reporting Summary

Nature Portfolio wishes to improve the reproducibility of the work that we publish. This form provides structure for consistency and transparency in reporting. For further information on Nature Portfolio policies, see our Editorial Policies and the Editorial Policy Checklist.

Statistics

For all statistical analyses, confirm that the following items are present in the figure legend, table legend, main text, or Methods section.

n/a	Confirmed	
☐	☒	The exact sample size (n) for each experimental group/condition, given as a discrete number and unit of measurement
☐	☒	A statement on whether measurements were taken from distinct samples or whether the same sample was measured repeatedly
☐	☒	The statistical test(s) used AND whether they are one- or two-sided<br>*Only common tests should be described solely by name; describe more complex techniques in the Methods section.*
☒	☐	A description of all covariates tested
☐	☒	A description of any assumptions or corrections, such as tests of normality and adjustment for multiple comparisons
☒	☐	A full description of the statistical parameters including central tendency (e.g. means) or other basic estimates (e.g. regression coefficient) AND variation (e.g. standard deviation) or associated estimates of uncertainty (e.g. confidence intervals)
☐	☒	For null hypothesis testing, the test statistic (e.g. F, t, r) with confidence intervals, effect sizes, degrees of freedom and P value noted<br>*Give P values as exact values whenever suitable.*
☒	☐	For Bayesian analysis, information on the choice of priors and Markov chain Monte Carlo settings
☒	☐	For hierarchical and complex designs, identification of the appropriate level for tests and full reporting of outcomes
☒	☐	Estimates of effect sizes (e.g. Cohen's d, Pearson's r), indicating how they were calculated

Our web collection on statistics for biologists contains articles on many of the points above.

Software and code

Policy information about availability of computer code

Data collection	Simulation results were obtained using R (version 4.1.2). The following open-source packages were used for this: deSolve (version 1.28; for integrating differential equations), mvtnorm (version 1.1-2; for multivariate Gaussian distributions), and tidyverse (version 1.3.1; for efficient data manipulation and plotting). The code is publicly available at https://github.com/dysordys/phenotypediv.
Data analysis	Both simulated and empirical data were analyzed using R (version 4.1.0 or higher). The following open-source packages were used for this: mvtnorm (version 1.1-2; for multivariate Gaussian distributions), and tidyverse (version 1.3.1; for efficient data manipulation and plotting), and ks (version 1.13.4; for multidimensional kernel density estimates). The code is publicly available at https://github.com/dysordys/phenotypediv.

For manuscripts utilizing custom algorithms or software that are central to the research but not yet described in published literature, software must be made available to editors and reviewers. We strongly encourage code deposition in a community repository (e.g. GitHub). See the Nature Portfolio guidelines for submitting code & software for further information.

Data

Policy information about availability of data

All manuscripts must include a data availability statement. This statement should provide the following information, where applicable:

- Accession codes, unique identifiers, or web links for publicly available datasets
- A description of any restrictions on data availability
- For clinical datasets or third party data, please ensure that the statement adheres to our policy

Raw data and metadata on the Galapagos land snail communities are publicly available at https://github.com/dysordys/phenotypediv.

Field-specific reporting

Please select the one below that is the best fit for your research. If you are not sure, read the appropriate sections before making your selection.

☐ Life sciences ☐ Behavioural & social sciences ☒ Ecological, evolutionary & environmental sciences

For a reference copy of the document with all sections, see nature.com/documents/nr-reporting-summary-flat.pdf

Ecological, evolutionary & environmental sciences study design

All studies must disclose on these points even when the disclosure is negative.

Study description	The study presents results from a mathematical model and uses an empirical dataset of the shell morphology of Galapagos land snail individuals. It argues that, over evolutionary timescales, functional trait diversity need not be positively related to species diversity.
Research sample	We use an empirical dataset of the shell morphology of Galapagos land snail individuals. Each row of the data holds information on one individual. The individuals were either sampled directly in the field, or were museum specimens that were measured. For every individual, the data summarize their species identity, whether the individual was found in arid or humid vegetation zones, and various measurements of their shell morphology (including centroid size and 28 PC axes of shell shape, with the first axis explaining over 80% of the variance in the data).
Sampling strategy	For each species we collected shell morphological measurements from up to 20 adult shells from each sampled locality. For extinct and otherwise difficult to sample species, we included as many adult shells as possible to best represent each respective species.
Data collection	Each individual snail shell was imaged via radiography by CP or AK. We then used the 'geomorph' R package to collect shell shape information from each shell.
Timing and spatial scale	Field sampling occurred from 2000-2015 as sampling opportunities permitted, and museum data collection occurred from 2000-2016.
Data exclusions	Three small satellite islands (CH, ED, and GA) were removed from the data, as they had no vegetation information. Additionally, there were only two sampled individuals of the species Naesiotus achatellinus, which does not allow for statistical inference. This species was therefore also removed.
Reproducibility	Individual shells are identified in the dataset such that measurements can be traced back to individual shells from museum collections or from the Parent Lab collection. Positions of individuals in shape morphospace are determined in part by the samples themselves, so shape variables are not directly comparable across experiments.
Randomization	Individuals are allocated to groups based on species, which were identified by CP and AK.
Blinding	Blinding was not possible during field and museum sampling as sample size limitations were set in part by the group identities (i.e. species).

Did the study involve field work? ☒ Yes ☐ No

Field work, collection and transport

Field conditions	Field conditions varied, but can best be described as tropical semi-arid
Location	Galapagos Islands, Ecuador
Access & import/export	Permits to access sites and to export samples were obtained from the Galapagos National Park Directorate.
Disturbance	Field collections followed the field work protocol established by the Galapagos National Park Directorate, which includes minimization of disturbance and collection of material restricted to approved permits.

Reporting for specific materials, systems and methods

We require information from authors about some types of materials, experimental systems and methods used in many studies. Here, indicate whether each material, system or method listed is relevant to your study. If you are not sure if a list item applies to your research, read the appropriate section before selecting a response.

Materials & experimental systems

n/a	Involved in the study
☒	☐ Antibodies
☒	☐ Eukaryotic cell lines
☒	☐ Palaeontology and archaeology
☒	☐ Animals and other organisms
☒	☐ Human research participants
☒	☐ Clinical data
☒	☐ Dual use research of concern

Methods

n/a	Involved in the study
☒	☐ ChIP-seq
☒	☐ Flow cytometry
☒	☐ MRI-based neuroimaging
